# Selective ablation of thymic and peripheral Foxp3^+^ regulatory T cell development

**DOI:** 10.3389/fimmu.2023.1298938

**Published:** 2023-12-18

**Authors:** Acelya Yilmazer, Dimitra Maria Zevla, Rikke Malmkvist, Carlos Alejandro Bello Rodríguez, Pablo Undurraga, Emre Kirgin, Marie Boernert, David Voehringer, Olivia Kershaw, Susan Schlenner, Karsten Kretschmer

**Affiliations:** ^1^ Molecular and Cellular Immunology/Immune Regulation, Center for Regenerative Therapies Dresden (CRTD), Center for Molecular and Cellular Bioengineering (CMCB), Technische Universität Dresden, Dresden, Germany; ^2^ Department of Infection Biology, Universitätsklinikum Erlangen and Friedrich-Alexander-Universität Erlangen-Nürnberg (FAU), Erlangen, Germany; ^3^ Department of Veterinary Medicine, Institute of Veterinary Pathology, Freie Universität Berlin, Berlin, Germany; ^4^ KU Leuven-University of Leuven, Department of Microbiology, Immunology and Transplantation, Leuven, Belgium; ^5^ Paul Langerhans Institute Dresden (PLID) of the Helmholtz Center Munich, University Hospital and Faculty of Medicine Carl Gustav Carus, Technische Universität Dresden, Dresden, Germany; ^6^ German Center for Diabetes Research (DZD e.V.), Neuherberg, Germany

**Keywords:** immune tolerance, autoimmunity, scurfy, diabetes, T cell development, pTreg, tTreg, Foxp3

## Abstract

Foxp3^+^ regulatory T (Treg) cells of thymic (tTreg) and peripheral (pTreg) developmental origin are thought to synergistically act to ensure immune homeostasis, with self-reactive tTreg cells primarily constraining autoimmune responses. Here we exploited a Foxp3-dependent reporter with thymus-specific GFP/Cre activity to selectively ablate either tTreg (ΔtTreg) or pTreg (ΔpTreg) cell development, while sparing the respective sister populations. We found that, in contrast to the tTreg cell behavior in ΔpTreg mice, pTreg cells acquired a highly activated suppressor phenotype and replenished the Treg cell pool of ΔtTreg mice on a non-autoimmune C57BL/6 background. Despite the absence of tTreg cells, pTreg cells prevented early mortality and fatal autoimmunity commonly observed in Foxp3-deficient models of complete Treg cell deficiency, and largely maintained immune tolerance even as the ΔtTreg mice aged. However, only two generations of backcrossing to the autoimmune-prone non-obese diabetic (NOD) background were sufficient to cause severe disease lethality associated with different, partially overlapping patterns of organ-specific autoimmunity. This included a particularly severe form of autoimmune diabetes characterized by an early onset and abrogation of the sex bias usually observed in the NOD mouse model of human type 1 diabetes. Genetic association studies further allowed us to define a small set of autoimmune risk loci sufficient to promote β cell autoimmunity, including genes known to impinge on Treg cell biology. Overall, these studies show an unexpectedly high functional adaptability of pTreg cells, emphasizing their important role as mediators of bystander effects to ensure self-tolerance.

## Introduction

The discovery of genetic *Foxp3* gene mutations as the culprit of the fatal autoimmune syndrome in the spontaneous *scurfy* mouse mutant (1, 2) and human IPEX patients (3, 4) provided the basis for unraveling the key role of Foxp3^+^ regulatory T (Treg) cells in dominant immunological tolerance. Observations in *Foxp3* gene-targeted mice further corroborated Treg cell paucity as the primary cause of early death and multi-organ autoimmunity in Foxp3-deficient mice (5) but also revealed the peripheral accumulation of Treg cell-like ‘wanna-be’ CD4^+^ T cells with self-reactive specificities (6–8) that contribute to the disease pathology (9, 10). Acute Foxp3^+^ Treg cell ablation recapitulated some, but not all aspects of the *scurfy* syndrome in non-autoimmune-prone mice (11, 12) and highlighted the continuous requirement of Treg cells to constrain organ-specific autoimmune responses in the spontaneous non-obese diabetic (NOD) mouse model of human type 1 diabetes (T1D) (13).

Since then, it has become clear that the physiologic Treg cell pool is developmentally heterogeneous (14–16), consisting of intrathymically (tTreg) and peripherally (pTreg) induced Treg cells that originate from distinct CD4^+^CD25^high^Foxp3^–^ precursor cells residing in thymus (17) and peripheral lymphoid tissues (18), respectively. In the thymus, distinct CD4^+^CD8^–^ single-positive (CD4SP) precursor cells, which exhibit low levels of Foxp3 protein preceding the up-regulation of CD25 expression, further expand the mature tTreg cell repertoire (19). In early studies examining the functional specialization of Treg cell developmental subsets by adoptive transfer immunotherapy of newborn *scurfy* mice (20), total Foxp3^+^ Treg cells prevented disease lethality, but did not suppress chronic inflammation and autoimmunity, which required the provision of Foxp3-sufficient CD4^+^ T cells to facilitate the extrathymic conversion of initially Foxp3^–^ T cells into functional Foxp3^+^ Treg cells (20). According to the prevailing view, tTreg cells are primarily positively selected by self-antigens during intrathymic development and are functionally specialized to control immune homeostasis and autoimmune responses (14, 21). The tTreg cell compartment in the spleen (SPL) and lymph nodes (LNs) has also been proposed to harbor Foxp3^+^ST2^+^ common precursors for tissue-type Treg cells (22) that accumulate and perform homeostatic and regenerative functions in nonlymphoid tissues (23), such as the visceral adipose tissue (24, 25). Consistent with tTreg cells as primary regulators of autoimmune responses, studies in mice with *Foxp3* gene-targeted deletion of conserved non-coding region 1 (CNS1) (Foxp3.CNS1^–/–^), which exhibit a significant, albeit incomplete block of pTreg cell development (26), failed to reveal severe autoimmune symptoms and have implicated pTreg cells in the control of immune responses at mucosal surfaces (27) and maternal-fetal tolerance (28). More recently, pTreg cells dependent on the gut microbiota have been shown to mediate functions beyond dominant suppression by facilitating muscle regeneration (29). With regard to a putative role of pTreg cells in the control of autoimmune responses, previous studies in the NOD model showed that dendritic cell (DC)-targeted self-antigen can encourage highly diabetogenic CD4^+^Foxp3^–^ T cells to acquire a Foxp3^+^ pTreg cell phenotype (30, 31), and that naturally induced, β cell-reactive pTreg cells are superior to tTreg cells with the same T cell receptor (TCR) specificity in constraining the manifestation of overt diabetes in a NOD.*Rag1* ^–/–^ adoptive transfer model (32). While Foxp3-deficient NOD mice failed to develop insulitis and overt diabetes (33), studies in Foxp3.CNS1^–/–^ NOD mice have provided ambiguous results, providing evidence for either a dispensable (34) or nonredundant function (35) of Foxp3.CNS1-dependent pTreg cells in the control of destructive β cell autoimmunity. In these studies, the relative contribution of tTreg cells to autoimmune β cell protection has not been directly addressed, owing to the lack of mouse models with selective tTreg cell paucity.

Here, we have exploited tTreg cell lineage-specific GFP/Cre recombinase activity in dual Foxp3^RFP/GFP^ reporter mice (32, 36) to generate complementary mouse lines that are deficient in either the tTreg (37) or pTreg cell lineage, while sparing the respective sister population. The results of subsequent loss-of-function studies revealed an unexpectedly high functional adaptability of naturally occurring pTreg cells in mice with selective tTreg cell paucity, thereby preventing the manifestation of severe *scurfy*-like symptoms commonly observed in mice with complete Treg cell deficiency. However, the acquisition of an increased genetic autoimmune risk associated with compromised Treg cell activity unleashed high mortality and a distinct pattern of autoimmune diseases, including severe β cell autoimmunity and overt diabetes.

## Materials and methods

### Selective *in vivo* ablation of developmental Treg cell sublineages

**Figure 1:**
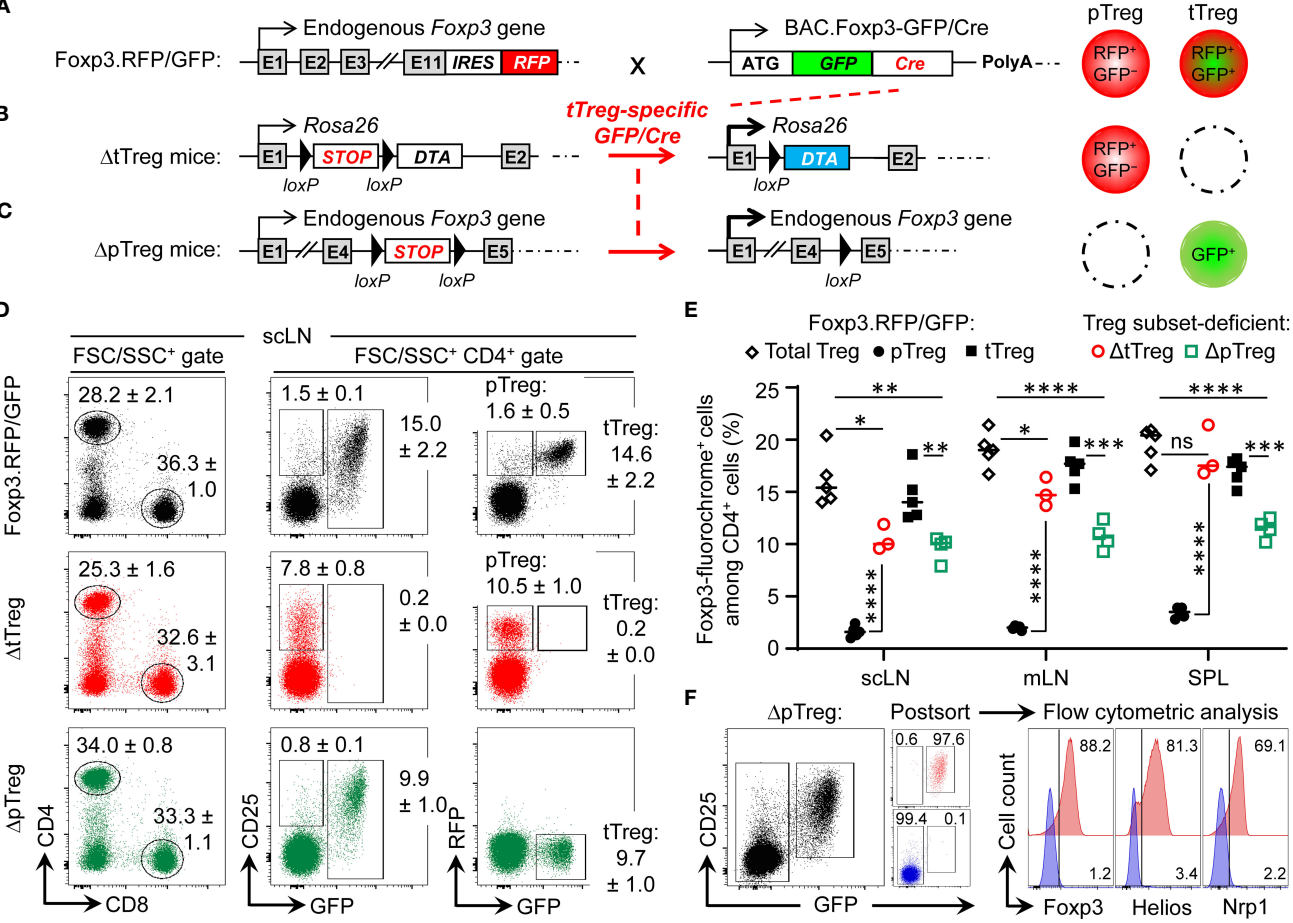
Selective ablation of tTreg and pTreg cell development *in vivo*. **(A–C)** Schematic overview of genetic strategy. **(A)** Foxp3^RFP/GFP^ mice. RFP is expressed from an IRES downstream of the endogenous *Foxp3* gene in both pTreg and tTreg cells. Restricted activation of BAC.Foxp3^GFP/Cre^ reporter expression to the thymus results in tTreg cell lineage-specific GFP/Cre activity and induction of gene expression by loxP-flanked STOP cassette excision. **(B)** ΔtTreg mice. Ablation of tTreg cells by GFP/Cre-mediated induction of diphtheria toxin A (DTA) expression. **(C)** ΔpTreg mice. A Foxp3.STOP cassette precludes pTreg cell development, while tTreg cell development can proceed after GFP/Cre-mediated induction of endogenous *Foxp3* gene expression. For this, the Foxp3^IRES-RFP^ reporter in **(A)** was replaced by a Cre-activatable Foxp3.STOP cassette. **(D–F)** Flow cytometry of Treg cells in peripheral lymphoid tissues. **(D)** Representative dot plots of (left) FSC/SSC-gated and (middle, right) CD4-gated cells from subcutaneous lymph nodes (scLNs) of 21-22-week-old males of indicated mouse lines. Numbers in dot plots represent mean percentages of cells ± SD within the respective gate. **(E)** Percentages of CD4-gated Foxp3-fluorochrome^+^ Treg cells in scLNs, mesenteric LNs (mLNs), and spleen (SPL) of Foxp3^RFP/GFP^ mice (pTreg: filled black circles, n = 5; tTreg: filled black squares, n = 5), ΔtTreg mice (pTreg: open red circles, n = 3), and ΔpTreg mice (tTreg: open green squares, n = 4). Note that the corresponding cell numbers are shown in **Supplementary Figure S1**. Symbols and horizontal lines represent individual mice and mean values, respectively. Unpaired t-test: ns, not significant; *p ≤ 0.05, **p ≤ 0.01, ***p ≤ 0.001, **** p ≤ 0.0001. **(F)** CD4^+^GFP^−^ T cells and CD4^+^CD25^+^GFP^+^ tTreg cells were FACS-purified from peripheral lymphoid organs of 3-5 males at 10 weeks of age and subjected to flow cytometric analysis of Foxp3, Helios, and Nrp1 expression after intracellular staining using fluorochrome-conjugated mAbs. Numbers in dot plots and histograms represent the percentage of cells within the respective gate.

Foxp3^RFP/GFP^ mice (**Figure 1A**) (32), congenic CD45.1 *scurfy* mice, and Rag2^–/–^ mice were on the C57BL/6 (B6) background. NOD.Foxp3^RFP/GFP^ mice were obtained by backcrossing B6.Foxp3^RFP/GFP^ mice onto the NOD/ShiLtJ background (Jackson Laboratories, Bar Harbor, USA) for ≥ 14 generations (32). For tTreg cell ablation (**Figure 1B**), B6.R26^DTA^ mice with Cre-activatable diphtheria toxin A (DTA) expression from the ubiquitous *Rosa26* gene locus (38) were crossed with B6.Foxp3^RFP/GFP^ mice (32, 37) or backcrossed to NOD.Foxp3^RFP/GFP^ mice, as indicated. For pTreg cell ablation (**Figure 1C**), a conditional Foxp3-STOP allele with Cre-activatable Foxp3 expression was crossed to B6.Foxp3^RFP/GFP^ mice, or backcrossed to NOD.Foxp3^RFP/GFP^ mice, as indicated. The Foxp3-STOP allele was developed at the University of Leuven (Genome Engineering Platform). In brief, a transcriptional STOP cassette, consisting of two loxP-flanked SV40 polyadenylation sites, was introduced by conventional gene targeting in E14 ES cells between exon 4 and 5 of the *Foxp3* gene, followed by excision of an FRT-flanked neomycin resistance gene using deleter mice [*Gt(ROSA)26Sor^tm1(FLP1)Dym^*/J; Stock No. 003946] (39). The conditional Foxp3-STOP allele was then backcrossed onto the B6 background for ≥ 10 generations and crossed with B6.Rag2^–/–^ mice, protecting B6.Foxp3-STOP mice from severe autoimmunity due to complete Foxp3^+^ Treg cell deficiency. All NOD mouse lines were fed with NIH #31M rodent diet (Altromin, Germany), and their blood glucose levels were routinely determined once a week using whole blood from the tail vein and Accu-Chek^®^ Aviva (Roche). Mice were considered diabetic at blood glucose levels above 200 mg/dl on at least two consecutive measurements or with blood glucose levels once above 400 mg/dl. All mice were housed and bred at the Animal Facility of the CRTD under specific pathogen-free conditions. Animal experiments were performed as approved by the Landesdirektion Dresden (25-5131/502/5, TVA 5/2020; 25-5131/522/43, TVV41/2021).

### Histopathology

After euthanizing the mice using CO_2_ inhalation, organs were collected and briefly washed in PBS. Subsequently, the tissues were fixed in a 4% paraformaldehyde solution (Sigma-Aldrich), paraffin-embedded, and 5 µm sections were cut. These sections were then stained with hematoxylin and eosin to assess histopathological changes. A total of 13 organs were examined (lung, heart, thymus, thyroid gland, stomach, liver, intestine, kidney, pancreas, urinary bladder, mesenteric adipose tissue, reproductive tract, brain) in a blinded manner to evaluate the presence and extent of inflammation and necrosis (none: 0; mild: +; moderate: ++; severe: +++) as described elsewhere (40).

### Flow cytometry and cell sorting

All single cell suspensions were prepared in Hank’s buffer (1×HBSS, 5% FCS, 10mM HEPES; all ThermoFisher, Life Technologies). For this, thymus (THY), spleen (SPL), mesenteric lymph nodes (mLN), pancreatic LN (pLN), and a pool of subcutaneous LN (scLN) (*Lnn. mandibularis, Lnn. cervicales superficiales, Lnn. axillares et cubiti, Lnn. inguinales superficiales*, and *Lnn. subiliaci*) were meshed through 70 μm cell strainers (BD Biosciences). Bone marrow (BM) cells were harvested from femurs and tibias by flushing mechanically dissociated bones or intact bone cavities with Hank’s buffer, followed by filtration through 70 μm cell strainers (BD). Single cell suspensions from SPL and BM were subjected to red blood cell lysis (erythrocyte lysis buffer, EL; Qiagen). Monoclonal antibodies (mAbs) to B220 (RA3-6B2), CD3ϵ (145-2C11), CD4 (RM4-5), CD8α (53-6.7), CD25 (PC61), CD62L (MEL-14), CD44 (IM7), CD45.1 (A20), CD45.2 (104), CD103 (M290), c-Kit (2B8), GITR (DTA-1), ICOS (7E.17G9), KLRG1 (2F1), PD-1 (29F.1A12), ST2 (U29-93), IgD (11-26c), IgM (II/41), MHC class II (I-A^b^: M5/114.15.2; I-A^G7^: OX-6), Foxp3 (FJK-16s), Helios (22F6), IL-10 (JES5-16E3), IFN-γ (XMG1.2), IL-17a (eBio17B7), IL-2 (JES6-5H4), IL-4 (11B11), IL-5 (TRFK5), TNF (MP6-XT22), Fc receptor-blocking mAb against CD16/32 (93), and fluorochrome-conjugated streptavidin (BUV395, eFlour450, APC and PE-Cy7) were purchased from BD, eBioscience, or Biolegend. Abs to Nrp1 (polyclonal goat IgG-AF700) were purchased from R&D Systems. Intracellular expression of cytokines and transcription factors was analyzed using the respective fluorochrome-coupled mAbs in conjunction with either the BD Cytofix/Cytoperm kit (BD) or the Foxp3 staining buffer set (eBioscience) according to the manufacturer’s protocol. The numbers of viable cells were determined using propidium iodide and a MACSQuant (Miltenyi Biotec). Before cell sorting, cells were enriched for CD4^+^ or CD25^+^ cells using biotinylated mAbs directed against CD4 or CD25, respectively, streptavidin-conjugated microbeads, fluorochrome-conjugated streptavidin, and the AutoMACS Pro magnetic cell separation system (Miltenyi Biotec). Samples were stained with DRAQ7 (BioStatus) for dead cell exclusion, filtered through 40 µm cell strainers, and analyzed on a LSR Fortessa or sorted using a FACS Aria II or III (all BD). Data were analyzed using FlowJo software (Version 10.8.1, Tree Star Inc.).

### Adoptive T cell transfer

Single cell suspensions from pooled LNs and SPL of B6.Foxp3^RFP/GFP^ donors (CD45.2) were subjected to CD4-based magnetic bead enrichment, followed by FACS-based isolation of total CD4^+^ T cells (i.e., including GFP^+^ tTreg cells) and CD4^+^GFP^−^ T cells (i.e., depleted of GFP^+^ tTreg cells). 1 x 10^7^ cells were injected i.p. into ≤ 2-day-old congenic CD45.1 *scurfy* recipient mice.

### T cell culture

T cells were cultured in 96-well round-bottom plates (Greiner) at 37°C and 5% CO_2_ in 200 μl RPMI complete medium [RPMI 1680 medium supplemented with 1 mM Sodium pyruvate, 1 mM HEPES, 2 mM Glutamax, 100 U/ml Penicillin, 100 µg/ml Streptomycin, 100 µg/ml Gentamycin, 0.1 mM non-essential amino acids, 0.55 mM β-mercaptoethanol and 10% FCS (v/v); all ThermoFisher, Life Technologies]. Prior to the flow cytometric analysis of intracellular cytokines, single cell suspensions were stimulated for 4 h in RPMI complete medium, using 50 ng/ml phorbol 12-myristate 13-acetate (PMA) and 200 ng/ml ionomycin (Iono), in the presence of 20 μg/ml Brefeldin A (all Merck, Sigma-Aldrich). For *in vitro* suppression, CD4^+^CD62L^high^CD25^−^Foxp3^−^ T responder (Tresp) cells and CD4^+^CD25^+^Foxp3^+^ Treg cells (RFP^+^GFP^+^ tTreg or RFP^+^GFP^−^ pTreg cells from B6.Foxp3^RFP/GFP^ mice; and GFP^+^ tTreg and RFP^+^GFP^−^ pTreg cells from B6 mice with selective pTreg and tTreg cell paucity, respectively) were FACS-isolated from peripheral lymphoid tissues. After labeling with the cell proliferation dye eFluor670 (5 µM, eBioscience), 5 × 10^4^ Tresp cells were cultured in triplicate wells with 10^5^ T cell-depleted splenocytes (magnetic bead depletion using anti-CD3, -CD4, and -CD8 mAb) and soluble anti-CD3ϵ mAb (0.5 μg/ml), either alone or with Treg cells at different Treg : Tresp cell ratios, as indicated. On day 3 after initiation of cultures, Tresp cell proliferation and CD25 expression was assessed by flow cytometry.

### Genomic PCR-based *Idd* gene analysis

Genomic DNA was isolated from tail biopsies using the NucleoSpin DNA RapidLyse kit (Macherey-Nagel) according to the manufacturer’s protocol. Genomic PCR was performed using DreamTag green DNA polymerase and buffer, dNTPs (all Thermo Fisher, Life Technologies), a set of 36 primer pairs (Eurofins Genomics) that cover most of the known *Idd* loci (see **Supplementary Materials and Methods** for a complete list) (41), and a Biometra Trio Thermocycler (Analytik Jena).

### Statistical analysis

Statistical significance was assessed using Prism 8 software (Version 8.4.3, GraphPad Software Inc., CA, USA). As indicated, the Student’s *t*-*test* (unpaired, two-tailed), Long-rank test (multiple comparisons with Bonferroni correction), and Chi-square test was used to assess statistical significance. Differences were considered as significant when *p ≤ 0.05, **p ≤ 0.01, ***p ≤ 0.001, ****p ≤ 0.0001.

## Results

### tTreg-specific Cre activity enables selective blockage of tTreg and pTreg cell development

In dual Foxp3^RFP/GFP^ reporter mice (**Figure 1A**), expression of the GFP/Cre fusion protein closely correlates with Foxp3 protein expression in the tTreg cell lineage, as transcriptional activation of the BAC.Foxp3^GFP/Cre^ reporter is restricted to the thymic *in vivo* environment (32, 36, 37, 42). In brief, the BAC.Foxp3^GFP/Cre^ reporter was completely inactive in physiologic Foxp3^–^CD4^+^CD25^+^ pTreg cell precursors at peripheral sites (18, 32), in various experimental settings of pTreg cell induction *in vivo*, and upon artificial Foxp3 induction in naïve CD4^+^Foxp3^–^ T cells *in vitro* (32). Accordingly, thymic Foxp3^–^CD25^+^ CD4SP tTreg cell precursors upregulated Foxp3-driven GFP/Cre expression during developmental progression *in situ*, or after intrathymic injection, but not *in vitro* in IL-2-supplemented cultures (32).

In Foxp3^RFP/GFP^ x R26^DTA^ mice (hereafter referred to as ΔtTreg mice) (37), GFP/Cre recombinase activity induces DTA expression selectively in the tTreg cell lineage by excision of an upstream loxP-flanked STOP cassette (38) (**Figure 1B**). DTA-mediated ablation (≥ 99.8%) resulted in the absence of RFP^+^GFP^+^ tTreg cells in scLN (**Figure 1D**) and at other peripheral sites, such as mesenteric LN and SPL (**Figure 1E**). A similarly high ablation efficiency was observed in peripheral lymphoid tissues of ΔtTreg mice with heterozygous (B6.R26^DTA^) and homozygous (B6.R26^DTA/DTA^) expression of the R26.STOP-DTA transgene, and over a wide age range (3-80 weeks; data not shown). As compared to Foxp3^RFP/GFP^ mice, selective tTreg cell paucity had no appreciable impact on the proportional distribution of CD4^+^ and CD8^+^ T cells in scLNs (**Figure 1D**, left), but was accompanied by a > 6-fold increase in the percentage of RFP^+^GFP^–^ pTreg cells among CD4-gated T cells (Foxp3^RFP/GFP^ mice: 1.6 ± 0.5%; ΔtTreg mice: 10.5 ± 1.0%) (**Figures 1D, E**; see **Supplementary Figure S1** for CD4^+^ T cell and Treg cell numbers). This marked increase in the pTreg cell population size of ΔtTreg mice largely compensated for the numerical impairment of the overall Treg cell pool in peripheral lymphoid tissues (**Figure 1E**; **Supplementary Figure S2A**), but to a significantly lesser extent in peripheral blood (**Supplementary Figure S2B**). In aged, > 1-year-old ΔtTreg mice, pTreg cells even exceeded total Treg cell frequencies of age-matched Foxp3^RFP/GFP^ mice (**Supplementary Figure S2C**).

To obtain mice with selective pTreg cell paucity (hereafter referred to as ΔpTreg mice), the Foxp3^IRES-RFP^ reporter of Foxp3^RFP/GFP^ mice was replaced by breeding with a Cre-activatable Foxp3.STOP cassette (**Figure 1C**). Hemizygous and homozygous Foxp3.STOP mice succumb to severe *scurfy* disease due to the absence of functional Foxp3 protein and Treg cells (43). In ΔpTreg mice, BAC.Foxp3^GFP/Cre^-mediated activation of Foxp3 expression selectively in tTreg cell lineage-committed thymocytes allowed for the formation of a robust peripheral GFP^+^ tTreg cell compartment (**Figures 1D, E**; **Supplementary Figure S1B**), while the extrathymic generation of pTreg cells remained precluded by the Foxp3.STOP cassette. Consistently, in peripheral lymphoid tissues of ΔpTreg mice, the expression of Foxp3, Helios, and Nrp1 was absent in FACS-purified CD4^+^GFP^–^ cells, but readily detectable in CD4^+^GFP^+^ tTreg cells (**Figure 1F**). Interestingly, selective pTreg cell deficiency resulted in a sustained reduction of the peripheral tTreg cell pool in adult (**Figure 1E**; **Supplementary Figure S2A**) and aged (**Supplementary Figure S2C**) ΔpTreg mice, consistent with previous observations in Foxp3.CNS1^−/−^ mice with impaired pTreg cell development (26, 27). These data, in conjunction with the ability of pTreg cells to compensate for the tTreg cell loss in ΔtTreg mice, suggest that pTreg cells are subject to less stringent constraints of the T cell receptor (TCR)-dependent clonal niche in peripheral lymphoid tissues, as compared to tTreg cells (44–46).

### Lymphopoiesis in ΔpTreg and ΔtTreg mice

**Figure 2:**
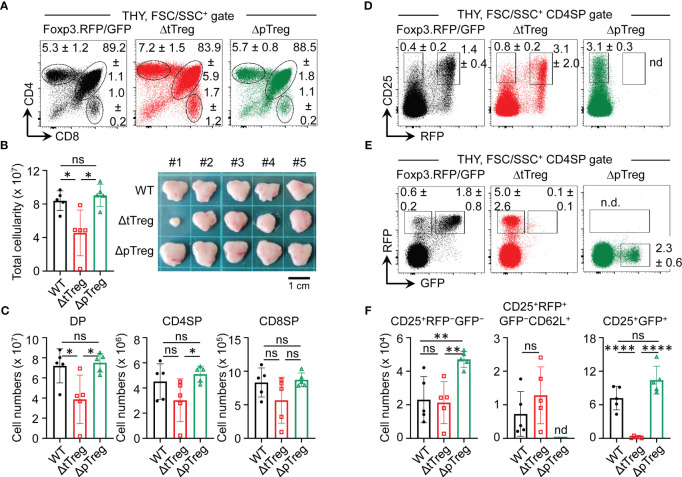
Thymopoiesis in adult ΔtTreg and ΔpTreg mice. Flow cytometry of thymic T cell development in Foxp3^RFP/GFP^, ΔtTreg, and ΔpTreg mice. **(A–C)** T cell development. **(A)** Representative flow cytometry of CD4 and CD8 expression among FSC/SSC-gated cells from the thymus (THY) of 17-22-week-old males, as indicated. **(B)** Total thymic cellularity (left) and thymus size (right). **(C)** Numbers of DP, CD4SP, and CD8SP cells. **(D–F)** tTreg cell development. Representative flow cytometry of **(D)** CD25 and Foxp3^IRES^-driven RFP expression, and **(E)** RFP and BAC. Foxp3^GFP/Cre^-driven GFP expression among gated CD4SP cells, as depicted in **(A)**. **(F)** Numbers of CD25^+^RFP^-^GFP^-^ (left), CD25^+^RFP^+^GFP^-^ (middle, pre-gated on CD62L^+^ cells to exclude mature recirculating CD62L^-^ Treg cells) and CD25^+^GFP^+^ (right) thymocytes. Note that ΔpTreg mice lack the Foxp3^IRES-RFP^ reporter (n.d., not detectable). Numbers in dot plots in **(A, D, E)** represent mean percentages of cells ± SD within the respective gate. Symbols and bars in **(B, C, F)** represent individual mice and mean values ± SD, respectively. Unpaired t-test: ns, not significant; *p ≤ 0.05, **p ≤ 0.01, ****p ≤ 0.0001. Data are from a single experiment (5 mice per group) representative of 4 experiments performed (3-6 mice per experiment).

Acute and chronic inflammatory immune responses are well-known to modulate hematopoietic activity, as exemplified by the manifestation of severe lympho-hematopoietic defects during ontogeny of *scurfy* mice (47–50). In the *scurfy* model, autoimmune-mediated thymic aberrations include severe post-developmental atrophy associated with enhanced apoptosis of CD4^+^CD8^+^ double-positive (DP) cells and concomitantly increased frequencies of CD4SP and CD8^+^ SP (CD8SP) cells (50). Unexpectedly, the proportional distribution of DP and SP cells in young (3-4-week-old; data not shown) and adult (13-22-week-old) (**Figure 2A**) ΔtTreg mice did not significantly differ from age-matched cohorts of ΔpTreg mice and Treg cell-proficient Foxp3^RFP/GFP^ mice. The average thymus size of adult ΔtTreg mice was only moderately reduced (< 2-fold; **Figures 2B, C**). We occasionally observed rare cases (≤ 10%) of severe thymic atrophy, which were restricted to individual ΔtTreg mice (**Figures 2B, C**) that additionally exhibited a markedly reduced body weight (ΔtTreg: mouse #1, 11.4 g; mouse #2-5, 28.4 ± 2.8 g; WT: 27.9 ± 1.4 g; ΔpTreg: 31.6 ± 4.1 g), while high frequencies of pTreg cells in peripheral lymphoid tissues remained unaffected (data not shown).

Next, we extended our observation of normal T lymphopoiesis in the majority of ΔtTreg mice to the analysis of B lymphopoiesis in the adult bone marrow (BM). In BM of *scurfy* mice, T cell-mediated autoimmune responses and systemically elevated levels of inflammatory cytokines have been shown to cause a complete block of B cell development, which is reflected by the absence of early Pro/Pre-B-I cell precursors and newly formed IgM^+^ B cells (47–50). However, our comparative flow cytometric analyses failed to reveal evidence for dysregulated B lymphopoiesis in ΔtTreg and ΔpTreg mice, as compared to Treg cell-proficient Foxp3^RFP/GFP^ mice (**Supplementary Figure S3**). This included comparable BM and SPL cellularity (**Supplementary Figure S3A**), as well as proportions and numbers of early B220^+^c-kit^+^ Pro/Pre-B-I precursor cells and immature B220^low^IgM^+^ B cells in BM (**Supplementary Figures S3B, C**), and of newly formed IgD^low^IgM^high^ B cells in the SPL (**Supplementary Figures S3D, E**).

### DTA-mediated tTreg cell ablation occurs prior to thymic exit

In the thymus of Foxp3^RFP/GFP^ reporter mice, tTreg cell lineage commitment induces the sequential expression of RFP and GFP in initially Foxp3^−^CD25^+^ CD4SP cells (32, 37). Specifically, the developmental progression of Foxp3^−^CD25^+^ CD4SP cells first initiates the simultaneous up-regulation of Foxp3 and RFP protein (giving rise to CD25^+^RFP^+^GFP^−^ cells) (**Figure 2D**; left panel), which is then followed by the timely delayed up-regulation of GFP/Cre expression, giving rise to newly formed Foxp3^+^CD25^+^ tTreg cells that are RFP^+^GFP^+^ (**Figure 2E**; left panel). In ΔpTreg mice, flow cytometry revealed no adverse effects of selective pTreg cell paucity on tTreg cell development (**Figures 2D, E**; right panels) and numbers of newly formed Foxp3^+^ tTreg cells (**Figure 2F**). In the thymus of ΔtTreg mice, tTreg cell development proceeded to the CD25^+^RFP^+^GFP^−^ CD4SP stage (**Figure 2D**; middle panel), but subsequent up-regulation of BAC.Foxp3^GFP/Cre^ reporter expression promoted DTA-mediated induction of apoptosis prior to thymic exit of newly formed CD25^+^RFP^+^GFP^+^ tTreg cells (**Figure 2E**; middle panel; **Figure 2F**). Thus, the observed deficiency in RFP^+^GFP^+^ tTreg cells at peripheral sites (**Figures 1D, E**) was already established within the thymus. Whereas, the proportional increase of CD4-gated RFP^+^GFP^−^ cells in ΔtTreg mice (**Figures 2D, E**) could be attributed to the intrathymic accumulation of mature pTreg cells (**Supplementary Figure S4A**) that originated from peripheral lymphoid tissues recirculating to the thymus, as indicated by heterogeneous CD25 expression levels and a ‘recirculating’ CD62L^−^CD69^+^CD44^high^ phenotype (**Supplementary Figure S4B**) (51, 52).

### pTreg cells prevent the early manifestation of severe autoimmunity in ΔtTreg mice

**Figure 3:**
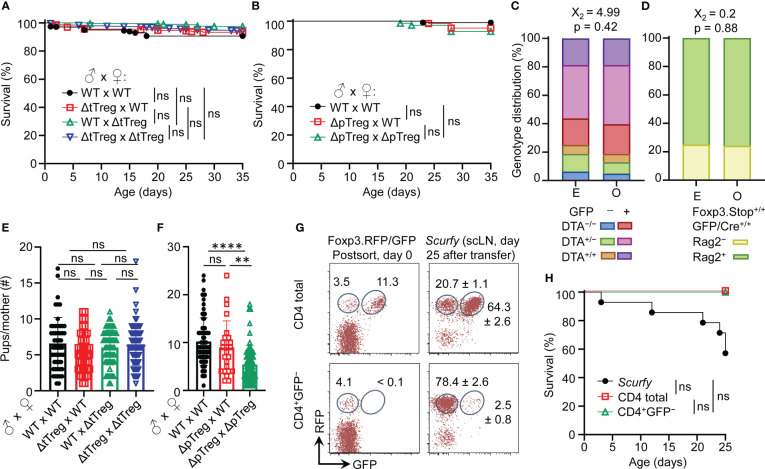
Viability and reproductive ability of ΔtTreg and ΔpTreg mice. **(A, B)** Kaplan-Meier survival curves of offspring produced by **(A)** ΔtTreg and **(B)** ΔpTreg pair mating. Newborn mice produced by indicated parental genotype combinations were monitored for the occurrence of spontaneous cases of mortality from birth onwards for up to 35 days (parental genotype, number of offspring; **(A)**: wild-type (WT) x WT, n = 342; ΔtTreg x WT, n = 227; WT x ΔtTreg, n = 398; ΔtTreg x ΔtTreg, n = 463. **(B)**: WT x WT, n = 103; ΔpTreg x WT, n = 62; ΔpTreg x ΔpTreg, n = 69). **(C, D)** Expected **(E)** and observed (O) distribution of offspring according to their genotype produced by **(C)** ΔtTreg (♂ DTA^+/−^GFP^+/−^ x ♀ DTA^+/−^GFP^+/−^; n = 187) and **(D)** ΔpTreg (♂ Rag2^+/−^Foxp3.Stop^+^GFP/Cre^+^ x ♀ Rag2^+/−^Foxp3.Stop^+/+^ GFP/Cre^+/+^; n = 62) pair mating. Chi-square test: X_2_, Chi-square. **(E, F)** The cumulative number of newborn pups produced by **(E)** ΔtTreg and **(F)** ΔpTreg pair mating of indicated parental genotype combinations. ΔtTreg: DTA^+/−^ or ^+/+^, BAC.Foxp3^GFP/Cre+^; ΔpTreg: Rag2^+/−^, Foxp3.Stop^hemi/homo^, BAC.Foxp3^GFP/Cre+^. Parental genotype, number of litters: **(E)**: WT x WT, n = 52; ΔtTreg x WT, n = 42; WT x ΔtTreg, n = 66; ΔtTreg x ΔtTreg, n = 72. **(F)**: WT x WT, n = 75; ΔpTreg x WT, n = 25; ΔpTreg x ΔpTreg, n = 60). Symbols and bars represent individual litters and mean values, respectively. **(G, H)** Adoptive Treg cell transfer into neonatal *scurfy* mice. **(G)** Flow cytometry of RFP and GFP expression among CD4-gated T cells before (post sort, left panels) and after (day 25, right panels) injection into conventional *scurfy* recipient mice. Numbers in dot plots represent percentages of cells (left) or mean percentages of cells ± SD (right) within the respective gate. **(H)** Kaplan-Meyer survival curves of *scurfy* mice that had either been left untreated (closed black circles, n = 14) or neonatally injected *i.p.* (5 x 10^5^ cells, day 0) with either total CD4^+^ T cells (open black squares, n = 4) or CD4^+^GFP^−^ T cells (open green triangles, n = 3) that had been FACS-purified from peripheral lymphoid tissues of Foxp3^RFP/GFP^ mice. At day 25, adoptively transferred CD4^+^CD45.2^+^ cells were tracked by flow cytometry in scLNs of congenic CD45.1^+^ recipient mice and analyzed for RFP and GFP expression. **(A, B, H)** Log-rank test and Bonferroni correction: ns, not significant. **(E, F)** Unpaired t-test: ns, not significant; **p ≤ 0.01, ****p ≤ 0.0001.

In our B6 *scurfy* colony maintained under specific-pathogen-free (SPF) conditions, approximately 50% of mice succumb to premature death by 35 days of age due to fatal autoimmunity associated with Foxp3 deficiency, and no mice live beyond 50 days (50). Within 35 days after birth, ΔtTreg (**Figure 3A**) and ΔpTreg (**Figure 3B**) mice appeared overall healthy, showing no appreciable spontaneous mortality (**Figures 3A, B**) or other *scurfy*-like symptoms (scaliness and crusting of eyelids/ears/tail, hepatomegaly, splenomegaly, lymphadenopathy, *etc.*) (data not shown). Consistently, our analysis of genotype distribution among 5-week-old mice revealed an unbiased heredity of the ΔtTreg (**Figure 3C**) and ΔpTreg (**Figure 3D**) phenotype.

The impaired generation of pTreg cells in Foxp3.CNS1^−/−^ mice has been reported to impinge on maternal-fetal tolerance by increasing the resorption of semiallogeneic fetuses (28). Our data show that the number of viable offspring produced by syngeneic ΔtTreg pair mating did not significantly differ from that of Foxp3^RFP/GFP^ mice (**Figure 3E**). In contrast, interstrain breeding of ΔpTreg mice gave rise to significantly reduced numbers of offspring, correlating with the ΔpTreg phenotype of the breeding female (**Figure 3F**). These findings in ΔtTreg and ΔpTreg mice, in conjunction with impaired implantation of syngeneic embryos after maternal Foxp3^+^ Treg cell depletion (53, 54), imply that pTreg cells may contribute to maternal-fetal tolerance even in syngeneic pregnancy.

The unexpected absence of severe *scurfy*-like symptoms in young B6.ΔtTreg mice suggested that selective tTreg cell paucity can be largely compensated by increased pTreg cell numbers (**Figures 1D, E**). We next asked whether the observed pTreg cell behavior in the ΔtTreg model can be recapitulated in *scurfy* mice neonatally injected with CD4^+^GFP^–^ T cell populations (including RFP^+^GFP^–^ pTreg cells) that had been FACS-isolated from peripheral lymphoid tissues of Foxp3^RFP/GFP^ mice for RFP^+^GFP^+^ tTreg cell depletion (**Figure 3G**, bottom left). In these experiments, total CD4^+^ T cells (including RFP^+^GFP^–^ pTreg and RFP^+^GFP^+^ tTreg cells) were included for comparison (**Figure 3G**, top left). Both cohorts of *scurfy* recipients were viable (**Figure 3H**) and appeared phenotypically healthy until the end of the observation period, apart from mild symptoms of delayed growth and exfoliative dermatitis in individual mice that received tTreg cell-depleted CD4^+^GFP^–^ T cells (data not shown). In the absence of tTreg cells, the adoptive CD4^+^GFP^–^ T cell transfer resulted in a marked accumulation of RFP^+^GFP^–^ pTreg cells among CD4^+^ T cells (day 0: 4.1%; day 25: 78.4 ± 2.6%) in *scurfy* recipients (**Figure 3G**, bottom panels), most likely due to both the proliferative expansion of preformed pTreg cells and the conversion of initially CD4^+^Foxp3^–^ T cells (20). In *scurfy* recipients of total CD4^+^ T cells, RFP^+^GFP^–^ pTreg cell frequencies among CD4-gated cells also increased (day 0: 3.5%; day 25: 20.7 ± 1.1%), but the initial pTreg:tTreg cell ratio of 1:3 was preserved (**Figure 3G**, top panels). Overall, these data indicate that pTreg cells can fill up the tTreg cell niche in both ΔtTreg (**Figures 1D, E**) and *scurfy* mice (**Figures 3G, H**) while maintaining a stable RFP^+^GFP^–^ phenotype.

### Maintenance of T cell homeostasis in adult ΔtTreg mice

**Figure 4:**
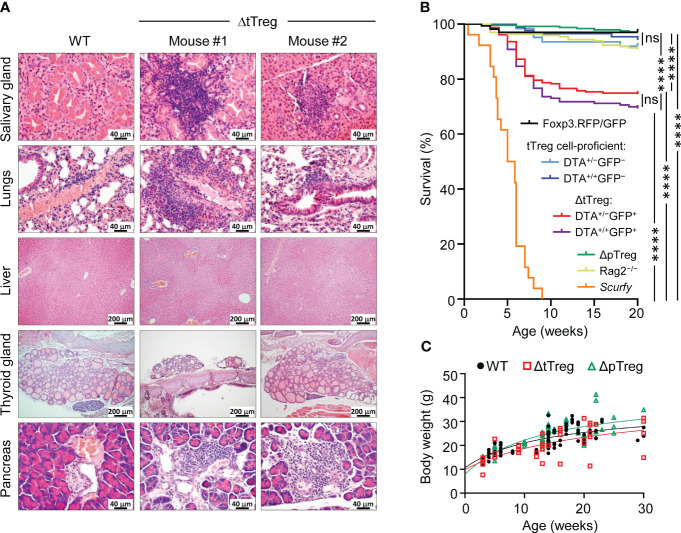
Autoimmune pathology and mortality of ΔtTreg mice. **(A)** Histological analysis. Organs of 4-week-old Foxp3^RFP/GFP^ (WT, left) and ΔtTreg (mouse #1 and #2) male mice were fixed in 4% PFA, and cut sections were stained with hematoxylin and eosin. In contrast to WT controls, individual ΔtTreg mice show mild leukocytic infiltrations in the salivary gland (mouse #1: +; mouse #2: ±), lungs (mouse #1: ++; mouse #2: ±), and pancreas (mouse #1: ++; mouse #2: ++). Sections from the liver and thyroid gland of WT and ΔtTreg mice lack leukocytic infiltrates and show normal organ structure. Magnifications: Salivary gland, lung, pancreas: 400x (bar = 40 µm); liver, thyroid gland: 100x (bar = 200 µm). **(B)** Kaplan-Meier survival analysis. Cohorts of Foxp3^RFP/GFP^ mice (n = 167), ΔtTreg mice (DTA^+/−^GFP^+^, n = 235; DTA^+/+^GFP^+^, n = 163), and ΔpTreg mice (n = 245) were monitored for the occurrence of spontaneous cases of mortality from birth onwards for up to 20 weeks, as indicated. R26^DTA^ mice lacking the BAC.Foxp3^GFP/Cre^ transgene (DTA^+/−^GFP^−^, n = 62; DTA^+/+^GFP^−^, n = 87), Foxp3-deficient *scurfy* mice (n = 26), and immunodeficient Rag2^-/-^ mice (n = 196) were included for comparison. Log-rank test and Bonferroni correction: ns, not significant; ****p ≤ 0.0001. **(C)** Age-dependent body weight gain of Foxp3^RFP/GFP^ (WT, closed black circles, n = 92), ΔtTreg (open red squares, n = 38), and ΔpTreg (open red triangles, n = 60) mice.

Our initial characterization of young ΔtTreg mice failed to reveal evidence for disease symptoms associated with selective tTreg cell paucity. Consistently, mild immune infiltrations were limited to the salivary gland (mouse #1), and the lung (mouse #1) of individual ΔtTreg mice, but could not be observed in other organs, such as the liver or thyroid gland (**Figure 4A**). Interestingly, although B6.ΔtTreg mice maintained normoglycemia, histological analyses consistently revealed pronounced immune infiltrates in the pancreas (**Figure 4A**, bottom panels), which is in contrast to previous studies in other settings of tTreg cell deficiency, including Foxp3-deficient mice (33) and acute Treg cell ablation in the ‘Depletion of Regulatory T cells’ (DEREG) mouse model on the B6 and NOD genetic background (13).

With the advancing age of ΔtTreg mice, we noticed rare cases of spontaneous deaths, which became first apparent at the age of 7 weeks (R26^DTA^: 18.7%, R26^DTA/DTA^: 19.0%; **Figure 4B**). The mortality of ΔtTreg mice further increased thereafter, reaching a plateau by 10 weeks that was maintained until the end of the 20-week observation period (R26^DTA^: 25.5%, R26^DTA/DTA^: 30.7%), remaining well below the high mortality of *scurfy* mice (**Figure 4B**). We rarely observed cases of spontaneous mortality among immunodeficient Rag2^–/–^ mice, or cohorts of tTreg cell-proficient, BAC.Foxp3^GFP/Cre–^ ΔtTreg littermates, ΔpTreg mice, and Foxp3^RFP/GFP^ mice (**Figure 4B**). We further noticed that mortality appeared to be associated in part with a reduced body weight and thymic atrophy of the affected ΔtTreg mice (**Figure 2B**). Our subsequent analyses showed that the majority of ΔtTreg mice had a body weight corresponding to their age (85.3%), but also confirmed that individual mice failed to keep up with physiological body weight gain (**Figure 4C**), while the small and large intestine yielded an unsuspicious histopathological result (data not shown).

**Figure 5:**
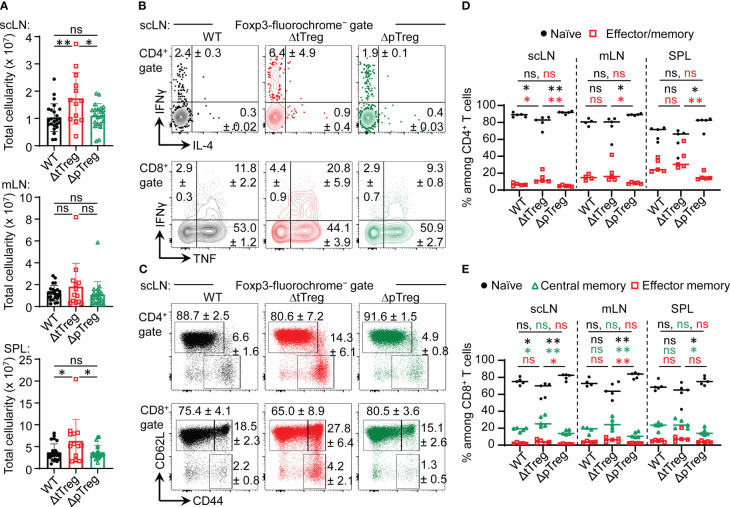
Peripheral T cell homeostasis in adult ΔtTreg and ΔpTreg mice. **(A)** Total cellularity of scLN (top), mLN, (middle), and SPL (bottom) of 13-22-week-old Foxp3^RFP/GFP^ (WT, n = 24), ΔtTreg (n = 13) and, ΔpTreg (n = 22) male mice. Symbols and bars represent individual mice and mean values ± SD, respectively. **(B–E)** Flow cytometry of CD4^+^ and CD8^+^ T effector cells in peripheral lymphoid tissues. **(B)** Representative dot plots of IFN-γ and IL-4 expression among gated CD4^+^Foxp3^−^ (top), and IFN-γ and IL-4 expression among gated CD8^+^ (bottom) T cells in scLN of 38-40-week-old WT (Foxp3^RFP/GFP^), ΔtTreg, and ΔpTreg male mice, as indicated (2-3 mice per group). **(C)** Representative dot plots of CD44 and CD62L expression among gated CD4^+^Foxp3^−^ (top) and CD8^+^ (bottom) T cells in scLN of 17-22-week-old WT (Foxp3^RFP/GFP^), ΔtTreg, and ΔpTreg male mice, as indicated (4-5 mice per group). (Gates: CD62L^high^CD44^low^, naïve; CD62L^low^CD44^high^, effector/memory; CD62L^high^CD44^high^, central memory). **(D, E)** Composite percentages of T cell effector subsets in 17-22-week-old Foxp3^RFP/GFP^ (WT), ΔtTreg, and ΔpTreg mice (4-5 male mice per group). **(D)** Naïve (CD62L^high^CD44^low^) and effector/memory (CD62L^low^CD44^high^) CD4^+^ T cell compartments. **(E)** Naïve (CD62L^high^CD44^low^), central memory (CD62L^high^CD44^high^), and effector/memory (CD62L^low^CD44^high^) CD8^+^ T cell compartments. Naïve: black closed circles; central memory: open green triangles; effector/memory: open red squares. Symbols and horizontal lines in **(A, D, E)** represent individual mice and mean percentages of cells ± SD, respectively. Numbers in dot plots in **(B, C)** indicate mean percentages of cells ± SD within the respective gate or quadrant. Unpaired t-test: ns, not significant; *p ≤ 0.05, **p ≤ 0.01.

With regard to peripheral T cell homeostasis in adult ΔtTreg mice, total cellularity of scLNs (but not of mLNs and SPL; see also **Supplementary Figure S3A**) was moderately, although significantly increased, as compared to Foxp3^RFP/GFP^ and ΔpTreg mice (**Figure 5A**). We only occasionally observed adult ΔtTreg mice with pronounced lymphadenopathy and splenomegaly (< 10%; **Figure 5A**). The analysis of inflammatory cytokine production indicated moderately increased proportions of IFN-γ- and IL-4-producing CD4^+^ T cells (**Figure 5B**, top), and of IFN-γ-producing CD8^+^ T cells (**Figure 5B**, bottom) in scLNs (but not in mLN or SPL; data not shown) of ΔtTreg mice. Increased expression of other cytokines (*e.g.*, IL-2, IL-10, IL-17) could also not be observed (data not shown). Consistent with largely normal frequencies of CD4^+^ and CD8^+^ T cells in the majority of ΔtTreg mice (**Figure 1D**), our flow cytometric analysis of CD62L and CD44 expression revealed no evidence for systemically uncontrolled activation of CD4^+^ and CD8^+^ T effector cells, neither in scLNs (**Figures 5C–E**) or other peripheral lymphoid tissues, such as mLN or SPL (**Figures 5D, E**).

### Compensatory adaptation of pTreg cell activity in the absence of tTreg cells

**Figure 6:**
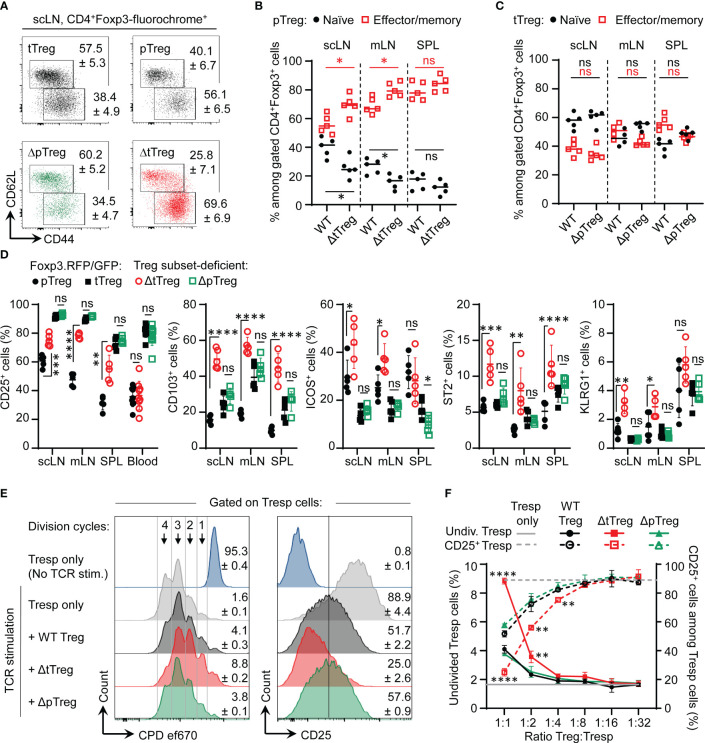
pTreg cell adaptation to tTreg cell paucity. **(A–C)** Flow cytometry of naïve and memory/effector-type Treg cell subsets. **(A)** Representative flow cytometry and cumulative percentages of **(B)** pTreg cells and **(C)** tTreg cells with a naïve (CD62L^high^CD44^low^, black filled circles) and effector/memory-type (CD62L^low^CD44^high^, red open squares) phenotype in peripheral lymphoid tissues (scLN, mLN, SPL) of ΔtTreg mice and ΔpTreg mice, respectively. Foxp3^RFP/GFP^ (WT) mice were included for comparison. Treg cell gating was as in **Figure 1D**. **(D)** Signature protein expression. Composite percentages of surface marker expression among Foxp3-fluorochrome reporter-gated CD4^+^ Treg cells (See **Supplementary Figure S5** for representative flow cytometry). Treg cell gating was as indicated in **Figure 1D**: pTreg (closed black circles) and tTreg cells (closed black squares) of Foxp3^RFP/GFP^ mice; pTreg cells of ΔtTreg mice (open red circles); and tTreg cells of ΔpTreg mice (open green squares). Symbols and horizontal lines indicate individual mice and mean values ± SD, respectively. Data are from a single experiment, representative of 4 independent experiments performed (n = 3-6 per group; age: 13-22 weeks). **(E, F)** Suppressor function *in vitro*. **(E)** Representative flow cytometry of T responder (Tresp) cell proliferation based on dilution of the proliferation dye (CPD) ef670 (left) and CD25 expression levels (right). Numbers in histograms indicate mean percentages of cells ± SD within the respective gate or quadrant. **(F)** Composite percentages of cell division (left, closed symbols) and CD25 expression (right, open symbols) of CD4^+^ Tresp cells at day 3 of co-culture, using total Treg cells of Foxp3^RFP/GFP^ mice (WT, black circles), pTreg cells of ΔtTreg mice (red squares), and tTreg cells (green triangles) of ΔpTreg mice at indicated Tresp : Treg ratios. For this, FACS-purified CD4^+^CD62L^+^Foxp3^−^CD25^−^ Tresp cells were co-cultured with APCs and 0.5 μg/ml α-CD3ϵ mAb, in the absence or presence of total Treg cells from scLN of Foxp3^RFP/GFP^ mice (WT, tTreg + pTreg), pTreg cells of ΔtTreg, or tTreg cells of ΔpTreg mice. Symbols and error bars in graphs indicate mean percentages ± SD of technical replicates (n = 2-3) from one experiment, representative of three independent experiments (5-10 mice per group at 20-22 weeks of age). Unpaired t-test: ns, not significant; *p ≤ 0.05, **p ≤ 0.01, ***p ≤ 0.001, ****p ≤ 0.0001.

Despite its already high percentage share in steady-state Foxp3^RFP/GFP^ mice (32), flow cytometric immunophenotyping (**Figure 6A**) indicated that the percentage of pTreg cells with a CD62L^low^CD44^high^ effector/memory-like phenotype further increased in LNs of adult ΔtTreg mice (**Figure 6B**), which was in contrast to tTreg cells in ΔpTreg (**Figure 6C**) mice. Consistent with an overall activated phenotype, pTreg cells in peripheral lymphoid tissues of ΔtTreg mice exhibited significantly upregulated expression of CD25 and several other ‘Treg cell signature’ proteins with functional relevance (including CD103, ICOS, ST2, and KLRG1), as compared to pTreg cells from Foxp3^RFP/GFP^ mice (**Figure 6D**; **Supplementary Figure S5**, top panels). In contrast, the reduced accumulation of pTreg cells in peripheral blood of ΔtTreg mice (**Supplementary Figure S2B**) was accompanied by low levels of CD25 expression (**Figure 6D**; left panel). Additionally, tTreg cells of ΔpTreg mice exhibited neither an increased effector/memory-like compartment (**Figure 6C**) nor up-regulated ‘Treg cell signature’ protein expression (**Figure 6D**; **Supplementary Figure S5**, bottom panels), as compared to their tTreg cell counterparts in Foxp3^RFP/GFP^ mice. Consistently, pTreg cells isolated from ΔtTreg mice suppressed the activity of Tresp cells more efficiently in standard cocultures, as judged by the inhibition of Tresp cell proliferation and CD25 expression, and as compared with tTreg cells from ΔpTreg mice or total Treg cells from Foxp3^RFP/GFP^ mice (**Figures 6E, F**). In summary, in contrast to tTreg cells of ΔpTreg mice, pTreg cells in peripheral lymphoid tissues (but not blood) of ΔtTreg mice acquire a highly activated state and increased suppressor function, which is indicative for their active involvement in constraining chronic immune dysregulation in the absence of tTreg cells (**Figure 4**).

### Fatal autoimmune pathology in ΔtTreg mice on a mixed (B6>NOD) background

**Figure 7:**
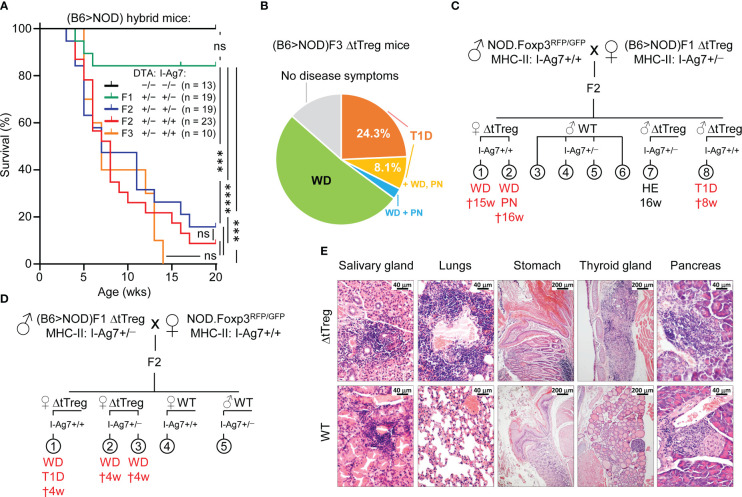
Spontaneous mortality and autoimmune pathology in ΔtTreg mice on a mixed (B6>NOD) background. B6.ΔtTreg mice were backcrossed to NOD.Foxp3^RFP/GFP^ mice for up to 3 generations, as indicated (F1, F2, F3). (B6>NOD) hybrid offspring were analyzed by flow cytometry for the haplotype-specific expression of MHC class II molecules (NOD: I-A^G7^; B6: I-A^b^). **(A)** Kaplan-Meier survival analysis. Cohorts of DTA^+/−^ ΔtTreg mice on a mixed (B6>NOD) background and with heterozygous I-Ag7^+/−^ (F1, n = 19; F2, n = 19) or homozygous I-Ag7^+/+^ (F2, n = 23; F3, n = 10) expression were monitored for the occurrence of spontaneous cases of mortality and morbidity from birth onwards for up to 20 weeks, as indicated. Note that tTreg cell-proficient B6.Foxp3^RFP/GFP^ mice (DTA^−/−^, I-Ag7^−/−^; n = 13) were included for comparison. Log-rank test and Bonferroni correction: ns, not significant; ***p ≤ 0.001, ****p ≤ 0.0001. **(B)** Morbidity of (B6>NOD)F3 ΔtTreg mice (37 males and females from 7 litters). WD: 51.4%, T1D: 24.3%, T1D + WD + PN: 8.1%, WD + PN: 2.7%, no disease symptoms: 13.5%. **(C, D)** Representative pedigrees of **(C)** (NOD>F1) and **(D)** (F1>NOD) ♂ x ♀ matings and health status of resultant F2 offspring. Parental (B6>NOD)F1 mice in **(B, C)** were obtained from independent (B6>NOD) backcross breedings. WD, wasting disease; T1D, type 1 diabetes; PN, peripheral neuropathy; HE, healthy (no disease symptoms); †: age of death in weeks. **(E)** Histological analysis. Organs of 11-13-week-old (B6>NOD)F3 males (I-Ag7^+/+^) were fixed in 4% PFA and cut sections were stained with hematoxylin and eosin. Representative histology showing pronounced leukocytic infiltrations and severe histopathological changes (score: +++) in the salivary gland, lungs, thyroid gland, stomach, and pancreas of a hyperglycemic R26^DTA^ NOD.ΔtTreg F3 mouse (top panels). R26^wt/wt^ NOD.Foxp3^RFP/GFP^ F3 mice (WT, bottom panels) show comparably severe immune infiltrates in the salivary glands, but only marginal (lungs, pancreas) or no (stomach, thyroid gland) autoimmune infiltration in other organs. Magnifications: Salivary gland, lung, pancreas: 400x (bar = 40 µm), stomach, thyroid gland: 100x (bar = 200 µm).

Our characterization of ΔtTreg mice on the B6 background revealed neither early- or late-onset of severe morbidity nor other signs of fatal autoimmunity (**Figures 3A**, **4A, B**) consistently observed in mice with complete Foxp3^+^ Treg cell deficiency (2, 5). However, the severity of autoimmune pathology associated with Treg cell deficiency can be markedly shaped by genetic factors: B6 *scurfy* mice can survive for up to 9 weeks after birth (**Figure 4B**), which is significantly longer than *scurfy* mice on the BALB/c background, all of which succumb to death within < 5 weeks of birth (20). Here, we aimed to explore how increased genetic susceptibility impinges on the survival and immune homeostasis of ΔtTreg mice by backcrossing the B6.ΔtTreg mouse line to autoimmune-prone NOD mice carrying the dual Foxp3^RFP/GFP^ reporter. On a pure NOD background, spontaneous T1D manifestation is under polygenic control of more than 20 insulin-dependent diabetes (*Idd*) gene loci (55), which additionally confer a broad susceptibility to multiple other autoimmune syndromes (peripheral neuropathy, autoimmune thyroiditis, *etc.*), albeit often with a low incidence (56). The analysis of (B6>NOD) hybrid mice obtained by two consecutive backcrosses (F2) revealed that selective tTreg cell paucity drastically decreased the survival of F2 ΔtTreg mice (both I-Ag7^+/–^ and I-Ag7^+/+^; see below) to ≤ 20% within 20 weeks after birth (**Figure 7A**), as compared to F1 ΔtTreg mice (84.2%; **Figure 7A**) and ΔtTreg mice on a pure B6 background (R26^DTA^: 74.5%, R26^DTA/DTA^: 69.3%; **Figure 4B**). Male and female F2 ΔtTreg mice showed no significant differences in mortality (data not shown). Continued backcrossing further exacerbated morbidity, such that none of the F3 ΔtTreg mice lived beyond 14 weeks (**Figure 7A**).

**Figure 8:**
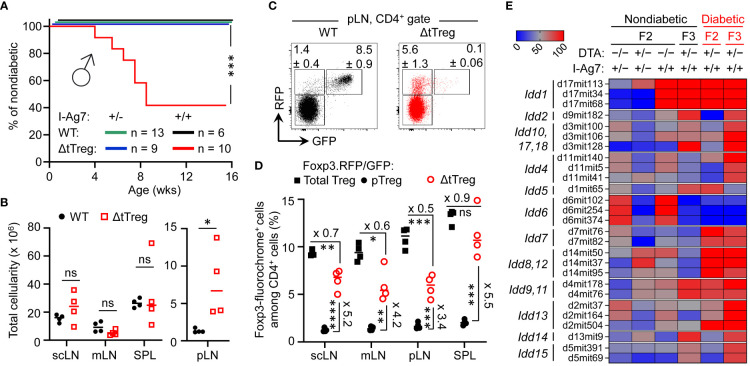
Pancreatic β cell autoimmunity in hybrid (B6>NOD) ΔtTreg mice. **(A)** Blood glucose levels of 3-week-old F2 ΔtTreg males with heterozygous (I-Ag7^+/−^, n = 9) or homozygous (I-Ag7^+/+^, n = 10) expression of I-Ag7 were monitored once a week. I-Ag7^+/−^ (n = 13) and I-Ag7^+/+^ (n = 6) F2 Foxp3^RFP/GFP^ mice (WT) mice were included for comparison. Note that initially normoglycemic mouse cohorts were selected based on the absence of WD, PN, or other *scurfy*-like symptoms. Log-rank test: ns, not significant; ***p ≤ 0.001. **(B–D)** Flow cytometric immunophenotyping of (B6>NOD)F2 mice depicted in **Figure 8A**. **(B)** Total cellularity of pLN (right) and other peripheral lymphoid tissues (scLN, mLN, SPL) of Foxp3^RFP/GFP^ (closed black circles) and ΔtTreg (open red squares) mice (all 8-week-old I-Ag7^+/+^ males). See **Supplementary Figure S6A** for corresponding numbers of CD8^+^ and CD4^+^ T cells. **(C)** Representative dot plots of Foxp3-driven RFP and GFP expression among CD4-gated cells from pLN from Foxp3^RFP/GFP^ and ΔtTreg mice, and **(D)** cumulative percentages of total Treg (closed black squares) and pTreg (closed black circles) cells of Foxp3^RFP/GFP^ mice, as well as pTreg cells of ΔtTreg mice (open red circles) from peripheral lymphoid tissues, as indicated (all I-Ag7^+/+^). Symbols and horizontal lines in **(B, D)** indicate individual mice and mean values of 4 mice per group, as depicted in **Figure 8A**. Unpaired t-test: ns, not significant; *p ≤ 0.05, **p ≤ 0.01, ***p ≤ 0.001, ****p ≤ 0.0001. **(E)** Genomic DNA-based *Idd* gene locus analysis in cohorts of (B6>NOD) hybrid mice. Cohorts of F2 Foxp3^RFP/GFP^ mice (WT; I-Ag7^+/−^: n = 17; I-Ag7^+/+^, n = 11); F2 ΔtTreg mice (I-Ag7^+/−^, n = 7; I-Ag7^+/+^, n = 5), and F3 ΔtTreg mice (nondiabetic I-Ag7^+/+^, n = 8; diabetic I-Ag7^+/+^, n = 5) were subjected to genomic PCR for *Idd* gene analysis. The heatmap shows the distribution of a selected set of *Idd* loci among different experimental groups, as indicated. For this, the percentage of mice homozygous for the respective NOD *Idd* gene locus was calculated and expressed as color code. An overview of the complete data set is provided in **Supplementary Figure S7**. Note that the R26-DTA transgene of ΔtTreg mice is embedded within the *Idd6* gene locus, resulting in a marked underrepresentation of *Idd6* in F2 and F3 ΔtTreg mice.

In order to account for possible variability of disease pathology on a mixed (B6>NOD) background, we produced independent cohorts of F2 and F3 ΔtTreg mice originating from unrelated (B6>NOD) backcross breedings and parental (B6>NOD)F1 mice (**Figures 7B–D**). When we monitored the ΔtTreg offspring for *scurfy*-like symptoms, we found that the high incidence of spontaneous mortality depicted in **Figure 7A** was consistently accompanied by signs of distinct, partially overlapping autoimmune diseases in independent cohorts of F2 ΔtTreg mice (**Figures 7C, D**). Most prominently, we observed signs of wasting disease (WD; reduced body weight and size, failure to thrive), autoimmune diabetes (hyperglycemia), and peripheral neuropathy (PN; hindlimb paralysis). In the (B6>NOD)F3 generation, ≥ 90% of ΔtTreg mice suffered from either WD (62.2%), T1D (32.4%), and/or PN (10.8%) (**Figure 7B**). Histopathological analyses indicated massive immune infiltrates of the salivary glands, the lung, the stomach, the thyroid glands, and the pancreas associated with severe tissue damage predominantly affecting thyroid glands and pancreatic islets of NOD.ΔtTreg F3 mice, which correlated with their hyperglycemic state (**Figure 7E**; top panel). A more detailed analysis of pancreatic islets (10 – 20 islets/mouse) revealed hardly any signs of immune infiltrates (mouse #1: 0%; mouse #2: < 10%; both non-diabetic) into the islets of tTreg cell-proficient (B6>NOD)F3 mice, whereas the majority of islets of ΔtTreg (B6>NOD)F3 mice (#1: > 90%, diabetic; #2: 70 – 80%; nondiabetic; #3: > 90%, diabetic) showed marked immune infiltrates (**Figure 7E**; data not shown). In these experiments, other organs commonly targeted by severe autoimmune responses in Foxp3-deficient mice showed no or only minimal immune infiltrates in NOD.ΔtTreg F3 mice, such as the small intestine or the liver (data not shown). Notably, severe salivary gland autoimmunity could be observed in both ΔtTreg and Foxp3^RFP/GFP^ mice (**Figure 7E**; left panels), and thus driven by the increased genetic autoimmune risk of the (B6>NOD)F3 background, rather than selective tTreg cell paucity. Other hallmarks of the fatal autoimmune syndrome affecting Foxp3-deficient mice could not be observed, such as skin lesions or scaliness and crusting of the eyelids, ears, and tail (data not shown). Throughout the present study, the manifestation of WD, T1D, and/or PN in tTreg cell-proficient littermates of (B6>NOD) hybrid ΔtTreg mice has also not been observed (**Figures 7B–E**, **8A**; and data not shown).

### Pancreatic β cell autoimmunity in (B6>NOD) hybrid ΔtTreg mice

While Foxp3^+^ Treg cell-deficient NOD mice fail to develop insulitis and overt diabetes (33), the data depicted in **Figure 7** provided the first indications that selective tTreg cell paucity can promote severe insulitis and overt diabetes (32.4%; **Figures 7B–E**) in both males and females, despite incomplete backcrossing onto the NOD background. However, more definite conclusions on the role of tTreg cells in controlling pancreatic β cell autoimmunity were hampered by the overall early onset of high morbidity and mortality (**Figure 7A**). In fact, three diabetic F3 NOD.ΔtTreg mice additionally exhibited signs of WD and PN (**Figure 7B**), suggesting that some (B6>NOD) hybrid ΔtTreg mice may succumb to death before the diabetes diagnosis. We therefore tracked blood glucose levels in cohorts of 3-week-old, initially normoglycemic F2 NOD.ΔtTreg mice that showed no signs of WD, PN, or other *scurfy*-like symptoms (**Figure 8A**). In our colony of conventional NOD mice, the first diabetes cases become apparent at approximately 12 weeks of age and continuously increase to an incidence of 70-90% in females and 0-20% in males within 30 weeks of age (13, 57). Whereas in (B6>NOD)F2 ΔtTreg mice, selective tTreg cell paucity unleashed a particularly severe form of T1D: > 50% of males rapidly progressed to overt diabetes within < 8 weeks after birth (**Figure 8A**), despite the usually observed female sex bias and kinetics difference in the NOD model (58). Flow cytometry-based MHC class II haplotyping indicated that diabetes manifestation in F2 ΔtTreg mice correlated with homozygous expression of the diabetogenic MHC class II molecule I-Ag7 of the NOD genetic background (*Idd1*), whereas mice co-expressing I-Ab of B6 origin remained normoglycemic during the observation period (**Figure 8A**). In contrast, high mortality (**Figure 7A**) and the manifestation of WD (**Figure 7D**) was independent of homozygous I-Ag7 expression.

In line with the absence of severe systemic autoimmune responses, pLNs of F2 ΔtTreg mice showed clear signs of lymphadenopathy, whereas the size of non-draining LNs (scLNs, mLNs) and SPL did not significantly differ between tTreg cell-deficient and -proficient (B6>NOD)F2 mice (**Figure 8B**). Consistently, numbers of CD8^+^ and CD4^+^ T cells (**Supplementary Figure S6A**, top panels) were selectively increased in pLNs of F2 ΔtTreg mice.

Consistent with our data in B6.ΔtTreg mice (**Figures 1D, E**), efficient intrathymic tTreg cell ablation in (B6>NOD)F2 ΔtTreg mice (**Supplementary Figure S6B**) was accompanied by a significant, up to 5.5-fold increase in the percentage of RFP^+^GFP^–^ pTreg cells among CD4-gated T cells in peripheral lymphoid tissues (**Figures 8C, D**; see **Supplementary Figure S6A** for Treg cell numbers). However, in contrast to B6.ΔtTreg mice (**Supplementary Figure S2A**), the population size of pTreg cells in (B6>NOD)F2 ΔtTreg mice only partially compensated for the numerical impairment of the overall Treg cell pool in the absence of tTreg cells (**Figure 8D**; **Supplementary Figure S6A**, bottom). Additionally, thymic cellularity (**Supplementary Figure S6C**) and numbers of T cell developmental stages (**Supplementary Figure S6D**) were consistently reduced in (B6>NOD)F2 ΔtTreg mice, as compared to in (B6>NOD)F2 Foxp3^RFP/GFP^ mice, probably due to increased hyperglycemia-induced stress and/or systemically elevated inflammatory cytokine levels.

### Contribution of NOD *Idd* loci to diabetes in (B6>NOD) hybrid ΔtTreg mice

We found that the manifestation of overt diabetes was restricted to I-Ag7^+/+^ (B6>NOD) hybrid ΔtTreg mice (**Figures 7B–E**, **8A**), consistent with the requirement of *Idd1* homozygosity for high penetrance of diabetes susceptibility in the NOD model (59). In fact, *Idd1* was shown to confer most of the diabetes risk (60), but not to be sufficient to precipitate diabetes in Foxp3^+^ Treg cell-proficient NOD mice (60). We hypothesized that the early manifestation of diabetes in I-Ag7^+/+^ F2 ΔtTreg males with high penetrance (**Figure 8A**) was driven by the acquisition of one or more additional, non-MHC-linked *Idd* loci. As expected, after only two backcross generations, PCR-based genomic *Idd* gene analysis (**Supplementary Figure S7**) indicated that the majority of *Idd* genes included in our survey was dispersible for diabetes development in I-Ag7^+/+^ F2 ΔtTreg mice (*Idd2*, *Idd3/Idd10/Idd17/Idd18*, *Idd4, Idd14, Idd15*) (**Supplementary Figure S7A**). This included *Il2* gene polymorphisms (encoded by *Idd3*), which play an important role in the reduced IL-2 receptor signaling strength received by Treg cells in conventional, tTreg cell-proficient NOD mice, resulting in their functional deficiency (61, 62). Additionally, *Idd6* was markedly underrepresented in (B6>NOD)F2 (**Supplementary Figure S7A**; **Figure 8E**) and F3 (**Supplementary Figure S7B** **;** **Figure 8E**) ΔtTreg mice, as compared to their Foxp3^RFP/GFP^ littermates, which can be attributed to the genomic localization of the R26-DTA transgene of ΔtTreg mice within the *Idd6* gene locus (http://www.informatics.jax.org/). Other *Idd* gene loci initially underrepresented in diabetic F2 ΔtTreg mice were found to be enriched after continued backcrossing (*Idd2, Idd4, Idd3/Idd10/Idd17/Idd18, Idd13.1/.2, Idd14, Idd15)* (**Supplementary Figure S7B**; **Figure 8E**).

In addition to *Idd1*, a set of 5 *Idd* gene loci (*Idd5.1*, *Idd7*, *Idd8/Idd12, Idd9.1/.2, Idd13.3*) was detectable in ≥ 80% of diabetic F2 ΔtTreg mice (**Figure 8E**), but was not sufficient to promote diabetes in tTreg cell-proficient I-Ag7^+/+^ (B6>NOD)F2 littermates (**Figure 8A**; and data not shown). Interestingly, this rather small set of *Idd* loci was primarily characterized by harboring genes with well-known functions in the development, survival/maintenance, function of pancreatic β cells [*Idd7*, *Idd8*, *Idd12* (63–67)] and Foxp3^+^ Treg cells (*Idd5*, *Idd7, Idd9.1/.2, Idd13.3*). In particular, several annotated genes located in *Idd5* [*Cd28*, *Icos*, *Ctla4* (68, 69); *Pdcd1* (70); *Irs1*, *Stat1* (71); *Ikfz2* (72)] and the *Idd9* gene locus [(*Cd30, Tnfr2, Cd137* (73, 74)*; p110δ, mTOR* (75, 76)] play key roles in various facets of Foxp3^+^ Treg cell biology (77–81), many of which belong to the shared transcriptional signatures of tissue-type Treg (22, 82, 83) and pTreg (32) cells. Furthermore, and directly relevant to the autoimmune mechanisms underlying pancreatic β cell destruction, *Idd7* (containing *Nfkbid*) has been implicated in modulating diabetogenic CD8^+^ T cell deletion in the thymus (84) and numbers and suppressor function of Foxp3^+^ Treg cells in peripheral tissues (85). Lastly, several annotated genes encoded in *Idd13* [*B2m* (86, 87)*; Mertk* (88)*; Bcl2l11/Bim, Id1, Smox, Pdia3* (89)] play key roles in development and activation of diabetogenic T effector cells (86, 87), including negative thymic selection (88–90).

**Figure 9:**
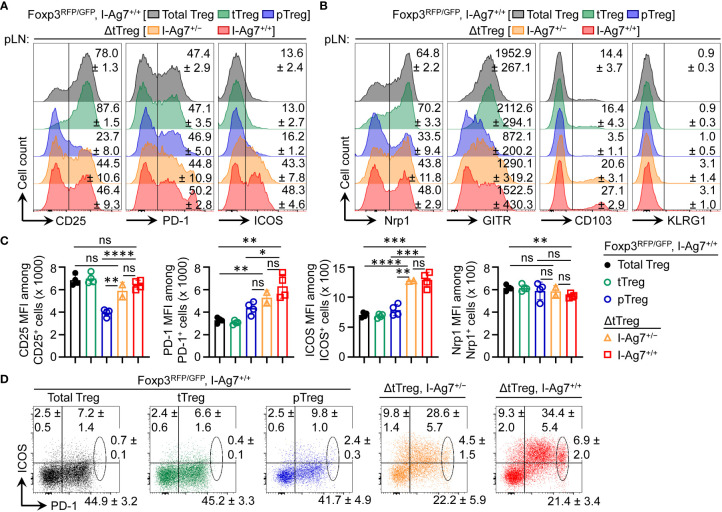
Flow cytometry-based immunophenotyping of pTreg cells in (B6>NOD)F2 ΔtTreg mice. Expression analysis of selected Treg cell signature proteins on gated Treg cell subsets of I-Ag7^+/+^ Foxp3^RFP/GFP^ mice (total Treg: grey; tTreg: green; pTreg: blue) and pTreg cells of ΔtTreg mice that were either I-Ag7^+/−^ (orange) or I-Ag7^+/+^ (red). Surface expression of **(A)** CD25, PD-1, and ICOS, and of **(B)** Nrp1, GITR, CD103, and KLRG1 on pTreg cells from F2 ΔtTreg mice, as compared to pTreg cells from F2 Foxp3^RFP/GFP^ mice. Numbers in histograms indicate mean percentages of cells ± SD within the respective gate or quadrant, with the exception of GITR in **(B)** showing mean fluorescence intensity (MFI) of the fluorochrome-conjugated mAb **(C)** Quantification of indicated marker expression based on MFI of fluorochrome-conjugated mAbs. Symbols and bars represent individual mice and mean values ± SD, respectively. **(D)** Expression of ICOS and PD-1 on pTreg cells from pLNs of Foxp3^RFP/GFP^ mice and ΔtTreg mice, as indicated. Numbers in dot plots indicate mean percentages of cells ± SD within the respective quadrant or gate. Note that gated populations of total Treg cells and tTreg cells from Foxp3^RFP/GFP^ mice were included for comparison. Data are from a single experiment (n = 4). Unpaired t-test: ns, not significant; *p ≤ 0.05, **p ≤ 0.01, ***p ≤ 0.001, ****p ≤ 0.0001.

Overall, these findings in ΔtTreg mice are consistent with a scenario, in which pTreg cell-mediated maintenance of immunological tolerance to pancreatic β cells can be abrogated by the acquisition of a limited set of *Idd* risk loci, some of which unfold their diabetogenic activity directly in pTreg cells. In support of this interpretation, comparative flow cytometry-based immunophenotyping revealed a correlation of some detected *Idd* loci and differential protein expression in pTreg cells of F2 ΔtTreg mice, as compared to pTreg cells in tTreg cell-proficient Foxp3^RFP/GFP^ littermates (**Figure 9**). This included the absence of *Idd3* (including *Il2*) and markedly increased expression levels of CD25 on pTreg cells from pLNs of diabetic F2 ΔtTreg mice, as compared to F2 Foxp3^RFP/GFP^ (**Figures 9A, C**; left panels). Relevant to their high diabetes susceptibility, the acquisition of *Idd5.1* (68) by F2 ΔtTreg mice correlated with increased expression levels of ICOS (*Icos*) and PD-1 (*Pdcd1*) on pTreg cells (**Figures 9A, C**), and the accumulation of an unusual ICOS^+^PD-1^high^ pTreg cell subset in F2 ΔtTreg mice, but not in Foxp3^RFP/GFP^ littermate controls (**Figure 9D**). In F2 ΔtTreg mice, other Treg signature proteins were either expressed on a higher proportion of pTreg cells (*e.g.*, Nrp1, CD103, KLRG1) or were expressed at higher levels (GITR) (**Figure 9B**). Importantly, functional incapacitation of PD-1 in gene-targeted mice (91–93) and human patients treated with blocking Abs (94) can result in overt autoimmune responses, including T1D (94–98), indicating a primarily inhibitory function of PD-1 expression in immune effector cells. However, independent lines of evidence in mice have pointed towards a diabetogenic role of PD-1 expression on Foxp3^+^ Treg cells, which included diabetes amelioration in congenic B10.Idd5^+^ and Idd5.1^+^ NOD mice (69, 99), diabetes protection of NOD mice with Foxp3^+^ Treg cell-specific PD-1 deletion (100), and an inverse correlation of PD-1 expression with the expression of Foxp3 and Foxp3^+^ Treg cell function (100, 101). Clearly, future studies are warranted to further dissect the differential function of PD-1 on T effector and Foxp3^+^ Treg cells, including pTreg cells.

## Discussion

As the functional heterogeneity of pTreg and tTreg cells promises to enable the subset-specific therapeutic manipulation of their activity in various clinical settings, it will be important to define their exact roles in establishing and maintaining peripheral immune homeostasis. The selective ablation of the development of pTreg cells (26, 27, 102) and tTreg cells, as done here, represents a considerable improvement over previous experiments relying on Foxp3-deficient mice and their reconstitution by adoptive Treg cell transfer, allowing the *in vivo* consequences to be analyzed under near-physiological conditions, including minimal autoimmune perturbations. In fact, some of the pathology observed in Foxp3-deficient mice has been attributed to the enhanced thymic export and peripheral accumulation of Treg cell-like ‘wanna-be’ CD4^+^ T cells with self-reactive specificities and distinct pathological properties (9, 10), rather than the mere absence of a functional Foxp3^+^ Treg cell pool. Additionally, some defects of Foxp3-deficient mice (*e.g.*, defective lympho-hematopoiesis) are refractory to adoptive Treg cell therapy, even when total CD4^+^ T cell populations were used (50). Here we have analyzed how selective tTreg cell paucity, which was achieved by intrathymic tTreg cell ablation while preserving pTreg cell generation, impinges on peripheral immune homeostasis in non-autoimmune and autoimmune-prone mice. Our data in B6.ΔtTreg mice reveal the ability of pTreg cells to establish immune homeostasis after birth, maintain immune tolerance in young mice, and constrain catastrophic autoimmune responses during aging in the majority of B6.ΔtTreg mice. Consistently, neonatal transfer of total CD4^+^ T cell populations, which had been depleted of tTreg cells, ameliorated clinical signs of Foxp3 deficiency in *scurfy* recipient mice. The manifestation of some mild disease symptoms (moderate growth delay and mild exfoliative dermatitis) can probably be attributed to an initial lag phase after tTreg cell-depleted CD4^+^ T cell transfer, which is required for seeding and proliferative expansion of pre-formed pTreg cells, and the lymphopenia-driven *de novo* generation of Foxp3^+^ pTreg cells (20).

Besides the absence of *scurfy*-like symptoms, several additional observations in the B6.ΔtTreg model further support our interpretation that physiologic pTreg cell populations can efficiently constrain autoimmune responses in the absence of tTreg cells. This includes an overall normal size of peripheral lymphoid tissues and T effector cell compartments (numbers, activation state, inflammatory cytokine production, etc.), as well as unperturbed lympho-hematopoiesis, representing a particularly sensitive indicator for the absence of ongoing (auto)immune responses. Many organs of B6.ΔtTreg mice, which are commonly targeted by severe autoimmune destruction in Foxp3-deficient mice, show no or only mild immune infiltrations not accompanied by any appreciable tissue destruction. Thus, the underlying cause promoting the occurrence of spontaneous deaths from an age of > 7 weeks onwards has remained less clear but may involve the exacerbation of chronic, low-level inflammation in individual organs, such as the thyroid gland or the lungs (**Figure 4A**), rather than multi-organ autoimmunity observed in Foxp3-deficient models of complete Treg cell deficiency. Interestingly, extending our histopathological analyses of the lungs (**Figure 4A**) to the upper respiratory tract of ΔtTreg mice that presented with reduced body weight provided first evidence for unexpected, severe inflammatory changes in the area of the nasal and oral cavities, pointing towards decreased food intake as a possible reason underlying a reduced body weight and morbidity of this particular disease subphenotype (data not shown).

Considering the age-dependent increase in spontaneous deaths (**Figure 4B**), the abrogation of immune homeostasis in individual ΔtTreg mice is likely to involve immunological and/or environmental cues (102–104), which are subject to age-related changes. This may include differences in the exposure to antigens derived from the diet and commensal microbiota promoting the physiologic induction of pTreg cells (103, 105–107). Our efforts to further analyze the immune events associated with the age-related impairment of peripheral immune homeostasis in ΔtTreg mice have been hampered by the relatively low incidence of mortality, in conjunction with rapid disease progression. In-depth flow cytometry-based immunophenotyping failed to reproducibly reveal age-related changes in the peripheral immune effector compartments of ΔtTreg mice, including CD4^+^ and CD8^+^ T effector compartments (**Figures 5C–E**). While this could be taken as an indication for quantitative and/or qualitative changes affecting the pTreg cell compartment, our analyses have not provided any evidence for an age-related reduction of pTreg cell numbers or phenotypic changes in the peripheral pTreg pool. In contrast, we found that the increased pTreg cell population size largely compensated for the numerical impairment of the overall Treg cell pool in adult ΔtTreg mice (**Figures 1D, E**; **Supplementary Figures S1B**, **S2**), which also holds true for ΔtTreg mice that were affected by reduced body weight and thymic atrophy (**Figure 2B**).

At present, we can only speculate on whether the pTreg cell niche in peripheral lymphoid tissues of ΔtTreg mice is replenished early in life and then maintained by proliferative expansion of pre-formed Treg cells, or whether continuous incorporation of newly formed cells is required to maintain a peripheral pTreg cell pool and immune homeostasis. This raises the possibility that the observed age-dependent increase in spontaneous mortality and morbidity is, at least in part, associated with a reduced efficiency in pTreg cell generation. In fact, pTreg cells are thought to be mainly, if not exclusively drawn from initially naïve CD4^+^ T cells (108). However, rates of thymic export of newly formed CD4^+^ T cells to peripheral sites of pTreg cell generation continuously decrease during aging and involution of the thymus (109, 110), but also during thymic atrophy due to chronic inflammatory stress (**Figure 2B**; **Supplementary Figure S6C**). Consistently, immediate CD25^high^Foxp3^−^ pTreg cell precursors residing in peripheral lymphoid tissues of nonmanipulated mice are strongly enriched among recent thymic emigrants (18).

Overall, our findings are consistent with a scenario, in which pTreg cells in peripheral lymphoid tissues of B6.ΔtTreg mice acquire a highly activated phenotype and increased suppressor function to cope with latent, chronic autoimmune responses due to the absence of tTreg cells. This intricate equilibrium can get out of control even by subtle age-related immunological and/or microenvironmental changes, which then tip the balance in favor of fatal autoimmunity. This may include changes in the commensal microbiota and qualitative differences among the pTreg cell pool, *e.g.*, reduced rates of pTreg cell *de novo* generation, in conjunction with proliferative pTreg cell expansion narrowing the TCR repertoire.

This interpretation was further corroborated by the dramatically increased mortality associated with the early onset of severe autoimmune diseases that could be observed in ΔtTreg mice after only two backcross generations onto the autoimmune-prone NOD background. Here we focused our analysis on pancreatic β cell autoimmunity, as T1D is considered a paradigmatic autoimmune disease for the application of Treg cell-based therapies to prevent or interfere with ongoing autoimmune destruction, although the main regulator(s) of pancreatic β cell autoimmunity hasn’t been identified yet. Lastly, Foxp3-deficient NOD mice with a polyclonal CD4^+^ T cell repertoire fail to present with insulitis and overt diabetes before they succumb at 3 weeks to severe inflammatory infiltration in multiple organs (33), precluding NOD.Foxp3-deficient mice as an experimental model to study the role of Treg cells in the autoimmune β cell protection. Our data show that the acquisition of a small set of *Idd* risk loci, many of which encode genes with well-known functions in Treg cell biology, is sufficient to precipitate a particularly severe form of autoimmune diabetes in ΔtTreg mice on a mixed (B6>NOD) background. In this context, it is of interest to note that our complementary studies in ΔpTreg mice on a (B6>NOD)F5 background haven’t provided evidence for a protective role of pTreg cells in the control of β cell autoimmunity (D.M.Z. and K.K., unpublished observation). Our observations in (B6>NOD) hybrid mice with selective tTreg cell paucity, in conjunction with previous experiments in Foxp3.CNS1^–/–^ with impaired pTreg cell development (34) indicate that tTreg cells are key regulators of β cell autoimmunity in the NOD model. Clearly, future experiments are warranted using Treg cell-subset-deficient mice on a pure NOD background to provide a more definite answer on the role of tTreg and pTreg cells in the control of β cell autoimmunity.

In conclusion, ΔtTreg and ΔpTreg mice offer to directly analyze the individual roles of tTreg and pTreg cells, respectively, in the control of immune homeostasis and organ-specific autoimmunity under near-physiologic conditions, which will facilitate future studies on the functional heterogeneity of the mature Treg cell pool. Besides autoimmune diseases, of particular interest will be to dissect their subset-specific contributions to non-immune functions that have recently been attributed to tissue-type Treg cells, which include facilitating homeostasis and regeneration of nonlymphoid tissues.

## Data availability statement

The original contributions presented in the study are included in the article/**Supplementary Material**. Further inquiries can be directed to the corresponding author.

## Ethics statement

The animal study was approved by Landesdirektion Dresden, Germany. The study was conducted in accordance with the local legislation and institutional requirements.

## Author contributions

AY: Writing – original draft, Conceptualization, Data curation, Formal analysis, Investigation, Methodology, Visualization. DZ: Writing – review & editing, Formal analysis, Investigation. RM: Writing – review & editing, Formal analysis, Investigation. CR: Writing – review & editing, Formal analysis, Investigation. PU: Writing – review & editing, Formal analysis, Investigation. EK: Writing – review & editing, Formal analysis, Investigation. MB: Writing – review & editing, Formal analysis, Investigation, Methodology, Project administration. DV: Writing – review & editing, Conceptualization, Formal analysis, Resources. OK: Writing – review & editing, Formal  analysis, Investigation, Methodology, Visualization. SS: Writing – original draft, Conceptualization, Formal analysis, Methodology, Resources, Supervision. KK: Writing – original draft, Conceptualization, Data curation, Formal analysis, Funding acquisition, Investigation, Methodology, Project administration, Resources, Supervision, Validation.

## References

[1] Chatila TA Blaeser F Ho N Lederman HM Voulgaropoulos C Helms C. JM2, encoding a fork head-related protein, is mutated in X-linked autoimmunity-allergic disregulation syndrome. J Clin Invest (2000) 106(12):R75–81. doi: 10.1172/JCI11679 PMC387260 11120765

[2] Brunkow ME Jeffery EW Hjerrild KA Paeper B Clark LB Yasayko SA. Disruption of a new forkhead/winged-helix protein, scurfin, results in the fatal lymphoproliferative disorder of the scurfy mouse. Nat Genet (2001) 27(1):68–73. doi: 10.1038/83784 11138001

[3] Bennett CL Christie J Ramsdell F Brunkow ME Ferguson PJ Whitesell L. The immune dysregulation, polyendocrinopathy, enteropathy, X-linked syndrome (IPEX) is caused by mutations of FOXP3. Nat Genet (2001) 27(1):20–1. doi: 10.1038/83713 11137993

[4] Wildin RS Ramsdell F Peake J Faravelli F Casanova JL Buist N. X-linked neonatal diabetes mellitus, enteropathy and endocrinopathy syndrome is the human equivalent of mouse scurfy. Nat Genet (2001) 27(1):18–20. doi: 10.1038/83707 11137992

[5] Fontenot JD Gavin MA Rudensky AY. Foxp3 programs the development and function of CD4+CD25+ regulatory T cells. Nat Immunol (2003) 4(4):330–6. doi: 10.1038/ni904 12612578

[6] Hsieh CS Zheng Y Liang Y Fontenot JD Rudensky AY. An intersection between the self-reactive regulatory and nonregulatory T cell receptor repertoires. Nat Immunol (2006) 7(4):401–10. doi: 10.1038/ni1318 16532000

[7] Lin W Haribhai D Relland LM Truong N Carlson MR Williams CB. Regulatory T cell development in the absence of functional Foxp3. Nat Immunol (2007) 8(4):359–68. doi: 10.1038/ni1445 17273171

[8] Lahl K Mayer CT Bopp T Huehn J Loddenkemper C Eberl G. Nonfunctional regulatory T cells and defective control of Th2 cytokine production in natural scurfy mutant mice. J Immunol (2009) 183(9):5662–72. doi: 10.4049/jimmunol.0803762 19812199

[9] Kuczma M Podolsky R Garge N Daniely D Pacholczyk R Ignatowicz L. Foxp3-deficient regulatory T cells do not revert into conventional effector CD4+ T cells but constitute a unique cell subset. J Immunol (2009) 183(6):3731–41. doi: 10.4049/jimmunol.0800601 PMC2771373 19710455

[10] Wyss L Stadinski BD King CG Schallenberg S McCarthy NI Lee JY. Affinity for self antigen selects Treg cells with distinct functional properties. Nat Immunol (2016) 17(9):1093–101. doi: 10.1038/ni.3522 PMC4994872 27478940

[11] Kim JM Rasmussen JP Rudensky AY. Regulatory T cells prevent catastrophic autoimmunity throughout the lifespan of mice. Nat Immunol (2007) 8(2):191–7. doi: 10.1038/ni1428 17136045

[12] Lahl K Loddenkemper C Drouin C Freyer J Arnason J Eberl G. Selective depletion of Foxp3+ regulatory T cells induces a scurfy-like disease. J Exp Med (2007) 204(1):57–63. doi: 10.1084/jem.20061852 17200412 PMC2118432

[13] Watts D Janßen M Jaykar M Palmucci F Weigelt M Petzold C. Transient depletion of foxp3(+) regulatory T cells selectively promotes aggressive β Cell autoimmunity in genetically susceptible DEREG mice. Front Immunol (2021) 12:720133. doi: 10.3389/fimmu.2021.720133 34447385 PMC8382961

[14] Sakaguchi S Yamaguchi T Nomura T Ono M. Regulatory T cells and immune tolerance. Cell (2008) 133(5):775–87. doi: 10.1016/j.cell.2008.05.009 18510923

[15] Lee HM Bautista JL Hsieh CS. Thymic and peripheral differentiation of regulatory T cells. Adv Immunol (2011) 112:25–71. doi: 10.1016/B978-0-12-387827-4.00002-4 22118406

[16] Shevach EM Thornton AM. tTregs, pTregs, and iTregs: similarities and differences. Immunol Rev (2014) 259(1):88–102. doi: 10.1111/imr.12160 24712461 PMC3982187

[17] Lio CWJ Hsieh CS. A two-step process for thymic regulatory T cell development. Immunity (2008) 28(1):100–11. doi: 10.1016/j.immuni.2007.11.021 PMC2248212 18199417

[18] Schallenberg S Tsai PY Riewaldt J Kretschmer K. Identification of an immediate Foxp3– precursor to Foxp3+ regulatory T cells in peripheral lymphoid organs of nonmanipulated mice. J Exp Med (2010) 207(7):1393–407. doi: 10.1084/jem.20100045 PMC2901063 20584884

[19] Owen DL Mahmud SA Sjaastad LE Williams JB Spanier JA Simeonov DR. Thymic regulatory T cells arise *via* two distinct developmental programs. Nat Immunol (2019) 20(2):195–205. doi: 10.1038/s41590-018-0289-6 30643267 PMC6650268

[20] Haribhai D Williams JB Jia S Nickerson D Schmitt EG Edwards B. A requisite role for induced regulatory T cells in tolerance based on expanding antigen receptor diversity. Immunity (2011) 35(1):109–22. doi: 10.1016/j.immuni.2011.03.029 PMC3295638 21723159

[21] Pohar J Simon Q Fillatreau S. Antigen-specificity in the thymic development and peripheral activity of CD4(+)FOXP3(+) T regulatory cells. Front Immunol (2018) 9:1701. doi: 10.3389/fimmu.2018.01701 30083162 PMC6064734

[22] Delacher M Imbusch CD Hotz-Wagenblatt A Mallm JP Bauer K Simon M. Precursors for nonlymphoid-tissue treg cells reside in secondary lymphoid organs and are programmed by the transcription factor BATF. Immunity (2020) 52(2):295–312.e11. doi: 10.1016/j.immuni.2019.12.002 31924477 PMC7026712

[23] Campbell C Rudensky A. Roles of regulatory T cells in tissue pathophysiology and metabolism. Cell Metab (2020) 31(1):18–25. doi: 10.1016/j.cmet.2019.09.010 31607562 PMC7657366

[24] Feuerer M Herrero L Cipolletta D Naaz A Wong J Nayer A. Lean, but not obese, fat is enriched for a unique population of regulatory T cells that affect metabolic parameters. Nat Med (2009) 15(8):930–9. doi: 10.1038/nm.2002 PMC3115752 19633656

[25] Cipolletta D Feuerer M Li A Kamei N Lee J Shoelson SE. PPAR-γ is a major driver of the accumulation and phenotype of adipose tissue Treg cells. Nature (2012) 486(7404):549–53. doi: 10.1038/nature11132 PMC3387339 22722857

[26] Zheng Y Josefowicz S Chaudhry A Peng XP Forbush K Rudensky AY. Role of conserved non-coding DNA elements in the Foxp3 gene in regulatory T-cell fate. Nature (2010) 463(7282):808–12. doi: 10.1038/nature08750 PMC2884187 20072126

[27] Josefowicz SZ Niec RE Kim HY Treuting P Chinen T Zheng Y. Extrathymically generated regulatory T cells control mucosal TH2 inflammation. Nature (2012) 482(7385):395–9. doi: 10.1038/nature10772 PMC3485072 22318520

[28] Samstein RM Josefowicz SZ Arvey A Treuting PM Rudensky AY. Extrathymic generation of regulatory T cells in placental mammals mitigates maternal-fetal conflict. Cell (2012) 150(1):29–38. doi: 10.1016/j.cell.2012.05.031 22770213 PMC3422629

[29] Hanna BS Wang G Galván-Peña S Mann AO Ramirez RN Muñoz-Rojas AR. The gut microbiota promotes distal tissue regeneration *via* RORγ(+) regulatory T cell emissaries. Immunity (2023) 56(4):829–846.e8. doi: 10.1016/j.immuni.2023.01.033 36822206 PMC10101925

[30] Kretschmer K Apostolou I Hawiger D Khazaie K Nussenzweig MC von Boehmer H. Inducing and expanding regulatory T cell populations by foreign antigen. Nat Immunol (2005) 6(12):1219–27. doi: 10.1038/ni1265 16244650

[31] Petzold C Schallenberg S Stern JNH Kretschmer K. Targeted antigen delivery to DEC-205^+^ dendritic cells for tolerogenic vaccination. Rev Diabetes Stud (2012) 9(4):305–18. doi: 10.1900/RDS.2012.9.305 PMC3740698 23804268

[32] Petzold C Steinbronn N Gereke M Strasser RH Sparwasser T Bruder D. Fluorochrome-based definition of naturally occurring Foxp3+ regulatory T cells of intra- and extrathymic origin. Eur J Immunol (2014) 44(12):3632–45. doi: 10.1002/eji.201444750 25159127

[33] Chen Z Herman AE Matos M Mathis D Benoist C. Where CD4+CD25+ T reg cells impinge on autoimmune diabetes. J Exp Med (2005) 202(10):1387–97. doi: 10.1084/jem.20051409 PMC2212985 16301745

[34] Holohan DR Van Gool F Bluestone JA. Thymically-derived Foxp3+ regulatory T cells are the primary regulators of type 1 diabetes in the non-obese diabetic mouse model. PloS One (2019) 14(10):e0217728. doi: 10.1371/journal.pone.0217728 31647813 PMC6812862

[35] Schuster C Jonas F Zhao F Kissler S. Peripherally induced regulatory T cells contribute to the control of autoimmune diabetes in the NOD mouse model. Eur J Immunol (2018) 48(7):1211–6. doi: 10.1002/eji.201847498 PMC6033626 29604048

[36] Schallenberg S Petzold C Tsai PY Sparwasser T Kretschmer K. Vagaries of fluorochrome reporter gene expression in Foxp3+ regulatory T cells. PloS One (2012) 7(8):e41971. doi: 10.1371/journal.pone.0041971 22879902 PMC3412838

[37] Simonetti M Yilmazer A Kretschmer K. Genetic tools for analyzing foxp3+ Treg cells: fluorochrome-based transcriptional reporters and genetic fate-mapping. BT - Regul T-Cells: Methods Protoc (2023) p:95–114. doi: 10.1007/978-1-0716-2647-4_8 36180629

[38] Voehringer D Liang HE Locksley RM. Homeostasis and effector function of lymphopenia-induced “memory-like” T cells in constitutively T cell-depleted mice. J Immunol (2008) 180(7):4742–53. doi: 10.4049/jimmunol.180.7.4742 PMC2670614 18354198

[39] Farley FW Soriano P Steffen LS Dymecki SM. Widespread recombinase expression using FLPeR (flipper) mice. Genesis (2000) 28(3–4):106–10. doi: 10.1002/1526-968X(200011/12)28:3/4<106::AID-GENE30>3.0.CO;2-T 11105051

[40] Telieps T Ewald F Gereke M Annemann M Rauter Y Schuster M. Cellular-FLIP, Raji isoform (c-FLIP R) modulates cell death induction upon T-cell activation and infection. Eur J Immunol (2013) 43(6):1499–510. doi: 10.1002/eji.201242819 23505065

[41] Serreze DV Chapman HD Varnum DS Hanson MS Reifsnyder PC Richard SD. B lymphocytes are essential for the initiation of T cell-mediated autoimmune diabetes: analysis of a new “speed congenic” stock of NOD.Ig mu null mice. J Exp Med (1996) 184(5):2049–53. doi: 10.1084/jem.184.5.2049 PMC2192892 8920894

[42] Junius S Mavrogiannis AV Lemaitre P Gerbaux M Staels F Malviya V. Unstable regulatory T cells, enriched for naïve and Nrp1(neg) cells, are purged after fate challenge. Sci Immunol (2021) 6(61):eabe4723. doi: 10.1126/sciimmunol.abe4723 34301799

[43] Gerbaux M Roos E Willemsen M Staels F Neumann J Bücken L. CTLA4-ig effectively controls clinical deterioration and immune condition in a murine model of foxp3 deficiency. J Clin Immunol (2023) 43(6):1393–402. doi: 10.1007/s10875-023-01462-2 PMC10354160 37156988

[44] Bautista JL Lio CWJ Lathrop SK Forbush K Liang Y Luo J. Intraclonal competition limits the fate determination of regulatory T cells in the thymus. Nat Immunol (2009) 10(6):610–7. doi: 10.1038/ni.1739 PMC2756247 19430476

[45] Leung MWL Shen S Lafaille JJ. TCR-dependent differentiation of thymic Foxp3+ cells is limited to small clonal sizes. J Exp Med (2009) 206(10):2121–30. doi: 10.1084/jem.20091033 PMC2757883 19737865

[46] Moran AE Holzapfel KL Xing Y Cunningham NR Maltzman JS Punt J. T cell receptor signal strength in Treg and iNKT cell development demonstrated by a novel fluorescent reporter mouse. J Exp Med (2011) 208(6):1279–89. doi: 10.1084/jem.20110308 PMC3173240 21606508

[47] Chen C Liu Y Liu Y Zheng P. Mammalian target of rapamycin activation underlies HSC defects in autoimmune disease and inflammation in mice. J Clin Invest (2010) 120(11):4091–101. doi: 10.1172/JCI43873 PMC2964994 20972332

[48] Leonardo SM Josephson JA Hartog NL Gauld SB. Altered B cell development and anergy in the absence of Foxp3. J Immunol (2010) 185(4):2147–56. doi: 10.4049/jimmunol.1000136 20639490

[49] Chang SE Guo L Tian J Liu Y Guo Z Zheng B. Autoimmune bone marrow environment severely inhibits B cell development by inducing extensive cell death and inhibiting proliferation. Autoimmunity (2012) 45(3):210–7. doi: 10.3109/08916934.2011.632455 22053866

[50] Riewaldt J Düber S Boernert M Krey M Dembinski M Weiss S. Severe developmental B lymphopoietic defects in foxp3-deficient mice are refractory to adoptive regulatory T cell therapy. Front Immunol (2012) 3:141. doi: 10.3389/fimmu.2012.00141 22679447 PMC3367401

[51] McCaughtry TM Wilken MS Hogquist KA. Thymic emigration revisited. J Exp Med (2007) 204(11):2513–20. doi: 10.1084/jem.20070601 PMC2118501 17908937

[52] Thiault N Darrigues J Adoue V Gros M Binet B Perals C. Peripheral regulatory T lymphocytes recirculating to the thymus suppress the development of their precursors. Nat Immunol (2015) 16(6):628–34. doi: 10.1038/ni.3150 25939024

[53] Kahn DA Baltimore D. Pregnancy induces a fetal antigen-specific maternal T regulatory cell response that contributes to tolerance. Proc Natl Acad Sci USA (2010) 107(20):9299–304. doi: 10.1073/pnas.1003909107 PMC2889122 20439708

[54] Heitmann RJ Weitzel RP Feng Y Segars JH Tisdale JF Wolff EF. Maternal T regulatory cell depletion impairs embryo implantation which can be corrected with adoptive T regulatory cell transfer. Reprod Sci (2017) 24(7):1014–24. doi: 10.1177/1933719116675054 PMC5933084 27834288

[55] Driver JP Serreze DV Chen YG. Mouse models for the study of autoimmune type 1 diabetes: a NOD to similarities and differences to human disease. Semin Immunopathol (2011) 33(1):67–87. doi: 10.1007/s00281-010-0204-1 20424843

[56] Aubin AM Lombard-Vadnais F Collin R Aliesky HA McLachlan SM Lesage S. The NOD mouse beyond autoimmune diabetes. Front Immunol (2022) 13:874769. doi: 10.3389/fimmu.2022.874769 35572553 PMC9102607

[57] Petzold C Riewaldt J Koenig T Schallenberg S Kretschmer K. Dendritic cell-targeted pancreatic beta-cell antigen leads to conversion of self-reactive CD4(+) T cells into regulatory T cells and promotes immunotolerance in NOD mice. Rev Diabetes Stud (2010) 7(1):47–61. doi: 10.1900/RDS.2010.7.47 PMC2923380 20703438

[58] Yurkovetskiy L Burrows M Khan AA Graham L Volchkov P Becker L. Gender bias in autoimmunity is influenced by microbiota. Immunity (2013) 39(2):400–12. doi: 10.1016/j.immuni.2013.08.013 PMC3822899 23973225

[59] Hattori M Buse JB Jackson RA Glimcher L Dorf ME Minami M. The NOD mouse: recessive diabetogenic gene in the major histocompatibility complex. Science (1986) 231(4739):733–5. doi: 10.1126/science.3003909 3003909

[60] Ikegami H Fujisawa T Sakamoto T Makino S Ogihara T. Idd1 and Idd3 are necessary but not sufficient for development of type 1 diabetes in NOD mouse. Diabetes Res Clin Pract (2004) 66 Suppl 1:S85–90. doi: 10.1016/j.diabres.2003.09.016 15563987

[61] Tang Q Adams JY Penaranda C Melli K Piaggio E Sgouroudis E. Central role of defective interleukin-2 production in the triggering of islet autoimmune destruction. Immunity (2008) 28(5):687–97. doi: 10.1016/j.immuni.2008.03.016 PMC2394854 18468463

[62] Long SA Cerosaletti K Wan JY Ho JC Tatum M Wei S. An autoimmune-associated variant in PTPN2 reveals an impairment of IL-2R signaling in CD4(+) T cells. Genes Immun (2011) 12(2):116–25. doi: 10.1038/gene.2010.54 PMC3058680 21179116

[63] Gao P Jiao Y Xiong Q Wang CY Gerling I Gu W. Genetic and molecular basis of QTL of diabetes in mouse: genes and polymorphisms. Curr Genomics (2008) 9(5):324–37. doi: 10.2174/138920208785133253 PMC2685644 19471607

[64] Itoh Y Kawamata Y Harada M Kobayashi M Fujii R Fukusumi S. Free fatty acids regulate insulin secretion from pancreatic beta cells through GPR40. Nature (2003) 422(6928):173–6. doi: 10.1038/nature01478 12629551

[65] Goulley J Dahl U Baeza N Mishina Y Edlund H. BMP4-BMPR1A signaling in beta cells is required for and augments glucose-stimulated insulin secretion. Cell Metab (2007) 5(3):207–19. doi: 10.1016/j.cmet.2007.01.009 17339028

[66] Hussain K. Insights in congenital hyperinsulinism. Endocr Dev (2007) 11:106–21. doi: 10.1159/000111066 17986831

[67] Schumann DM Maedler K Franklin I Konrad D Størling J Böni-Schnetzler M. The Fas pathway is involved in pancreatic beta cell secretory function. Proc Natl Acad Sci USA (2007) 104(8):2861–6. doi: 10.1073/pnas.0611487104 PMC1815272 17299038

[68] Greve B Vijayakrishnan L Kubal A Sobel RA Peterson LB Wicker LS. The diabetes susceptibility locus Idd5.1 on mouse chromosome 1 regulates ICOS expression and modulates murine experimental autoimmune encephalomyelitis. J Immunol (2004) 173(1):157–63. doi: 10.4049/jimmunol.173.1.157 15210770

[69] Wicker LS Chamberlain G Hunter K Rainbow D Howlett S Tiffen P. Fine mapping, gene content, comparative sequencing, and expression analyses support Ctla4 and Nramp1 as candidates for Idd5.1 and Idd5.2 in the nonobese diabetic mouse. J Immunol (2004) 173(1):164–73. doi: 10.4049/jimmunol.173.1.164 15210771

[70] Simpfendorfer KR Strugnell RA Brodnicki TC Wijburg OLC. Increased autoimmune diabetes in pIgR-deficient NOD mice is due to a “Hitchhiking” interval that refines the genetic effect of Idd5.4. PloS One (2015) 10(4):e0121979. doi: 10.1371/journal.pone.0121979 25835383 PMC4383422

[71] Ligons DL Guler ML Li HS Rose NR. A locus on chromosome 1 promotes susceptibility of experimental autoimmune myocarditis and lymphocyte cell death. Clin Immunol (2009) 130(1):74–82. doi: 10.1016/j.clim.2008.06.015 18951849 PMC2640841

[72] Hunter K Rainbow D Plagnol V Todd JA Peterson LB Wicker LS. Interactions between Idd5.1/Ctla4 and other type 1 diabetes genes. J Immunol (2007) 179(12):8341–9. doi: 10.4049/jimmunol.179.12.8341 18056379

[73] Lyons PA Hancock WW Denny P Lord CJ Hill NJ Armitage N. The NOD Idd9 genetic interval influences the pathogenicity of insulitis and contains molecular variants of Cd30, Tnfr2, and Cd137. Immunity (2000) 13(1):107–15. doi: 10.1016/S1074-7613(00)00012-1 10933399

[74] Forsberg MH Foda B Serreze DV Chen YG. Combined congenic mapping and nuclease-based gene targeting for studying allele-specific effects of Tnfrsf9 within the Idd9.3 autoimmune diabetes locus. Sci Rep (2019) 9(1):4316. doi: 10.1038/s41598-019-40898-8 30867509 PMC6416332

[75] Hamilton-Williams EE Rainbow DB Cheung J Christensen M Lyons PA Peterson LB. Fine mapping of type 1 diabetes regions Idd9.1 and Idd9.2 reveals genetic complexity. Mamm Genome (2013) 24(9–10):358–75. doi: 10.1007/s00335-013-9466-y PMC3824839 23934554

[76] Ferreira C Palmer D Blake K Garden OA Dyson J. Reduced regulatory T cell diversity in NOD mice is linked to early events in the thymus. J Immunol (2014) 192(9):4145–52. doi: 10.4049/jimmunol.1301600 24663675

[77] Sharpe AH Freeman GJ. The B7–CD28 superfamily. Nat Rev Immunol (2002) 2(2):116–26. doi: 10.1038/nri727 11910893

[78] Bour-Jordan H Bluestone JA. Regulating the regulators: costimulatory signals control the homeostasis and function of regulatory T cells. Immunol Rev (2009) 229(1):41–66. doi: 10.1111/j.1600-065X.2009.00775.x 19426214 PMC2714548

[79] Chapman NM Chi H. mTOR signaling, Tregs and immune modulation. Immunotherapy (2014) 6(12):1295–311. doi: 10.2217/imt.14.84 PMC4291176 25524385

[80] Chougnet C Hildeman D. Helios-controller of Treg stability and function. Transl Cancer Res (2016) 5(Suppl 2):S338–41. doi: 10.21037/tcr.2016.07.37 PMC6333417 30656143

[81] Gianchecchi E Fierabracci A. Inhibitory receptors and pathways of lymphocytes: the role of PD-1 in treg development and their involvement in autoimmunity onset and cancer progression. Front Immunol (2018) 9:2374. doi: 10.3389/fimmu.2018.02374 30386337 PMC6199356

[82] Delacher M Imbusch CD Weichenhan D Breiling A Hotz-Wagenblatt A Träger U. Genome-wide DNA-methylation landscape defines specialization of regulatory T cells in tissues. Nat Immunol (2017) 18(10):1160–72. doi: 10.1038/ni.3799 PMC5912503 28783152

[83] Miragaia RJ Gomes T Chomka A Jardine L Riedel A Hegazy AN. Single-cell transcriptomics of regulatory T cells reveals trajectories of tissue adaptation. Immunity (2019) 50(2):493–504.e7. doi: 10.1016/j.immuni.2019.01.001 30737144 PMC6382439

[84] Presa M Racine JJ Dwyer JR Lamont DJ Ratiu JJ Sarsani VK. A hypermorphic nfkbid allele contributes to impaired thymic deletion of autoreactive diabetogenic CD8(+) T cells in NOD mice. J Immunol (2018) 201(7):1907–17. doi: 10.4049/jimmunol.1800465 PMC6143397 30127089

[85] Dwyer JR Racine JJ Chapman HD Quinlan A Presa M Stafford GA. Nfkbid overexpression in nonobese diabetic mice elicits complete type 1 diabetes resistance in part associated with enhanced thymic deletion of pathogenic CD8 T cells and increased numbers and activity of regulatory T cells. J Immunol (2022) 209(2):227–37. doi: 10.4049/jimmunol.2100558 PMC9365269 35760520

[86] Serreze DV Bridgett M Chapman HD Chen E Richard SD Leiter EH. Subcongenic analysis of the Idd13 locus in NOD/Lt mice: evidence for several susceptibility genes including a possible diabetogenic role for beta 2-microglobulin. J Immunol (1998) 160(3):1472–8. doi: 10.4049/jimmunol.160.3.1472 9570569

[87] Hamilton-Williams EE Serreze DV Charlton B Johnson EA Marron MP Mullbacher A. Transgenic rescue implicates beta2-microglobulin as a diabetes susceptibility gene in nonobese diabetic (NOD) mice. Proc Natl Acad Sci USA (2001) 98(20):11533–8. doi: 10.1073/pnas.191383798 PMC58764 11572996

[88] Wallet MA Flores RR Wang Y Yi Z Kroger CJ Mathews CE. MerTK regulates thymic selection of autoreactive T cells. Proc Natl Acad Sci USA (2009) 106(12):4810–5. doi: 10.1073/pnas.0900683106 PMC2660755 19251650

[89] Liston A Hardy K Pittelkow Y Wilson SR Makaroff LE Fahrer AM. Impairment of organ-specific T cell negative selection by diabetes susceptibility genes: genomic analysis by mRNA profiling. Genome Biol (2007) 8(1):R12. doi: 10.1186/gb-2007-8-1-r12 17239257 PMC1839132

[90] Dugas V Liston A Hillhouse EE Collin R Chabot-Roy G Pelletier AN. Idd13 is involved in determining immunoregulatory DN T-cell number in NOD mice. Genes Immun (2014) 15(2):82–7. doi: 10.1038/gene.2013.65 24335706

[91] Salama AD Chitnis T Imitola J Ansari MJI Akiba H Tushima F. Critical role of the programmed death-1 (PD-1) pathway in regulation of experimental autoimmune encephalomyelitis. J Exp Med (2003) 198(1):71–8. doi: 10.1084/jem.20022119 PMC2196082 12847138

[92] Kroner A Schwab N Ip CW Ortler S Göbel K Nave KA. Accelerated course of experimental autoimmune encephalomyelitis in PD-1-deficient central nervous system myelin mutants. Am J Pathol (2009) 174(6):2290–9. doi: 10.2353/ajpath.2009.081012 PMC2684193 19443704

[93] Wang C Dehghani B Li Y Kaler LJ Vandenbark AA Offner H. Oestrogen modulates experimental autoimmune encephalomyelitis and interleukin-17 production *via* programmed death 1. Immunology (2009) 126(3):329–35. doi: 10.1111/j.1365-2567.2008.03051.x PMC2669813 19302141

[94] Tucker CG Dwyer AJ Fife BT Martinov T. The role of programmed death-1 in type 1 diabetes. Curr Diabetes Rep (2021) 21(6):20. doi: 10.1007/s11892-021-01384-6 PMC8323125 33956235

[95] Ansari MJI Salama AD Chitnis T Smith RN Yagita H Akiba H. The programmed death-1 (PD-1) pathway regulates autoimmune diabetes in nonobese diabetic (NOD) mice. J Exp Med (2003) 198(1):63–9. doi: 10.1084/jem.20022125 PMC2196083 12847137

[96] Wang J Yoshida T Nakaki F Hiai H Okazaki T Honjo T. Establishment of NOD-Pdcd1-/- mice as an efficient animal model of type I diabetes. Proc Natl Acad Sci USA (2005) 102(33):11823–8. doi: 10.1073/pnas.0505497102 PMC1188011 16087865

[97] Keir ME Liang SC Guleria I Latchman YE Qipo A Albacker LA. Tissue expression of PD-L1 mediates peripheral T cell tolerance. J Exp Med (2006) 203(4):883–95. doi: 10.1084/jem.20051776 PMC2118286 16606670

[98] Paterson AM Brown KE Keir ME Vanguri VK Riella LV Chandraker A. The programmed death-1 ligand 1:B7-1 pathway restrains diabetogenic effector T cells *in vivo* . J Immunol (2011) 187(3):1097–105. doi: 10.4049/jimmunol.1003496 PMC3148082 21697456

[99] Lamhamedi-Cherradi SE Boulard O Gonzalez C Kassis N Damotte D Eloy L. Further mapping of the Idd5.1 locus for autoimmune diabetes in NOD mice. Diabetes (2001) 50(12):2874–8. doi: 10.2337/diabetes.50.12.2874 11723074

[100] Tan CL Kuchroo JR Sage PT Liang D Francisco LM Buck J. PD-1 restraint of regulatory T cell suppressive activity is critical for immune tolerance. J Exp Med (2021) 218(1):e20182232. doi: 10.1084/jem.20182232 33045061 PMC7543091

[101] Wong M La Cava A Hahn BH. Blockade of programmed death-1 in young (New Zealand Black x New Zealand White)F1 mice promotes the suppressive capacity of CD4+ regulatory T cells protecting from lupus-like disease. J Immunol (2013) 190(11):5402–10. doi: 10.4049/jimmunol.1202382 PMC3700538 23636058

[102] Campbell C Dikiy S Bhattarai SK Chinen T Matheis F Calafiore M. Extrathymically generated regulatory T cells establish a niche for intestinal border-dwelling bacteria and affect physiologic metabolite balance. Immunity (2018) 48(6):1245–57.e9. doi: 10.1016/j.immuni.2018.04.013 29858010 PMC6260932

[103] Sefik E Geva-Zatorsky N Oh S Konnikova L Zemmour D McGuire AM. MUCOSAL IMMUNOLOGY. Individual intestinal symbionts induce a distinct population of RORγ^+^ regulatory T cells. Science (2015) 349(6251):993–7. doi: 10.1126/science.aaa9420 PMC4700932 26272906

[104] Russler-Germain EV Rengarajan S Hsieh CS. Antigen-specific regulatory T-cell responses to intestinal microbiota. Mucosal Immunol (2017) 10(6):1375–86. doi: 10.1038/mi.2017.65 PMC5939566 28766556

[105] Lathrop SK Bloom SM Rao SM Nutsch K Lio CW Santacruz N. Peripheral education of the immune system by colonic commensal microbiota. Nature (2011) 478(7368):250–4. doi: 10.1038/nature10434 PMC3192908 21937990

[106] Arpaia N Campbell C Fan X Dikiy S van der Veeken J deRoos P. Metabolites produced by commensal bacteria promote peripheral regulatory T-cell generation. Nature (2013) 504(7480):451–5. doi: 10.1038/nature12726 PMC3869884 24226773

[107] Kim KS Hong SW Han D Yi J Jung J Yang BG. Dietary antigens limit mucosal immunity by inducing regulatory T cells in the small intestine. Science (2016) 351(6275):858–63. doi: 10.1126/science.aac5560 26822607

[108] Schallenberg S Petzold C Riewaldt J Kretschmer K. Regulatory T cell-based immunotherapy: prospects of antigen-specific tolerance induction. In: Medical advancements in aging and regenerative technologies: clinical tools and applications. Hershey, Pennsylvania: IGI Global (2013). p. 112–36.

[109] Hale JS Boursalian TE Turk GL Fink PJ. Thymic output in aged mice. Proc Natl Acad Sci (2006) 103(22):8447–52. doi: 10.1073/pnas.0601040103 PMC1482512 16717190

[110] Fink PJ Hendricks DW. Post-thymic maturation: young T cells assert their individuality. Nat Rev Immunol (2011) 11(8):544–9. doi: 10.1038/nri3028 PMC3241610 21779032

[111] Yilmazer A Zevla DM Malmkvist R Rodríguez CAB Undurraga P Kirgin E. Selective ablation of thymic and peripheral Foxp3+ regulatory T cell development. bioRxiv [preprint] (2023). Available at: uri https://www.biorxiv.org/content/10.1101/2023.08.04.551974v1. 10.3389/fimmu.2023.1298938 PMC10757929 38164128

