# Supplementary material for: Selective ablation of thymic and peripheral Foxp3+ regulatory T cell development
Source: Front Immunol. 2023 Dec 18;14:1298938. doi: 10.3389/fimmu.2023.1298938 (PMC10757929; doi:10.3389/fimmu.2023.1298938)
Supplement: Supplementary file 1 [file DataSheet_1.pdf]

Supplementary Material

Selective ablation of thymic and peripheral Foxp3$^+$ regulatory T cell development

Acelya Yilmazer, Dimitra Maria Zevla, Rikke Malmkvist, Carlos Alejandro Bello Rodríguez, Pablo Undurraga, Emre Kirgin, Marie Boernert, David Voehringer, Olivia Kershaw, Susan Schlenner*, and Karsten Kretschmer*

*** Correspondence:** Susan Schlenner: susan.schlenner@kuleuven.be
Karsten Kretschmer: karsten.kretschmer@tu-dresden.de

Supplementary Materials and Methods

The following primers were used for Idd loci analyses:

d17mit113: 5'-TCT GTC TCC TCC GTA CTG GG, 5'-GTC AAT AAG TTC AAT CAC TGA ACA CA;

d17mit34: 5'-TGT TGG AGC TGA ATA CAC GC, 5'-GGT CCT TGT TTA TTC CCA GTA CC;

d17mit68: 5'-GTC CTG ACA TCA TGC TTT GTG, 5'-CTA CCG TTT GGA AGG CTG AG;

d9mit91: 5'-TTC TGG GGC AGA GAC CAG, 5'AGA AGG TGG CAG GGG TAG TT;

d9mit208: 5'-GCC TCT CTT TCT TTA AAC ACT TTA AG, 5'-CCT CCA CAC ACC TGT TTG TG;

d9mit182: 5'-GTG AAA TTG GTT ATG TAA ATG TCT GA, 5'-GAG ATG ACT AGG GTG AAC TGG G;

d3nds6: 5'-GTG GGA GTG TGT GCA AAA GAC, 5'-CAG AAT AGG TGA TTA GGT GGT TAT;

d3mit100: 5'-CCT GAT GAC TCT GCG TGT GT, 5'-ATA CCA GTG TTC TCC CCA ACC;

d3mit106: 5'-ACT TGT GCA TGG TGT GTA TGC, 5'-TGT GAT GGC ACC TTT GGT AA;

d3mit128: 5'-AAT AAA GGA AGA TGT CAT CTC AGT ACA, 5'-GAT GGG ATG GGA TGG GAT;

d11mit140: 5'-GCA TTT ACT TGA TTG ATT GTT TGC, 5'-ACC CAA TGC CTG CCT CTA C;

d11mit5: 5'-TTC TGT GAG CCT GGA GGA GT, 5'TAC AGG ACT AGT TTC CAT TTG GG;

d11mit41: 5'-CTG CTA AAG TGG GGT TAA ATG C, 5'-CGA CTG AGC AAG TTG TAT TTC TG;

d1mit65: 5'-CTA ACC CCT ATA CAC ATA CTG CCC, 5'-CCG TTC AGA CTT GAA TAC AGA CC;

d1mit18: 5'-TCT GGT TCC AGG CTT GAT TC, 5'-TCA CAA GTG AGG CTC CAG G;

d1mit8: 5'-CTG AAA ATC GTC CCT TGA CC, 5'-CAG GAG CAT GAA ATG GGG AT;

d1mit30: 5'-TGA ACC ATC ACC ATG CTG TT, 5'-TGG GCT GCG TTT CTA AGG;

d6mit102: 5'-CCA TGT GGA TAT CTT CCC TTG, 5'-GTA TAC CCA GTT GTA AAT CTT GTG TG;

d6mit254: 5'-AGT GTC CCT AGG GGG TGG, 5'-GGG GCC TTA GAG GTA GCA AC;

d6mit374: 5'-TTC TGG CTC TTA ACA GTC TGT CC, 5'-TAC ATA TGC CAA TGA TAT TCT CCC;

d7mit76: 5'-CAT GAG CAC GTG GAG AAA GA, 5'-CGT GGA AAC CTG ATA AAC TGA;

d7mit82: 5'-GGA CAC GGT GTC CAT CAA G, 5'-CTG AGT AGA AAG CAT GTG GGG;

d14mit50: 5-'GAG GGG GAA TCC TAG TGC TC, 5'-AGC AAA GCC CTA TCC ACA TG;

d14mit37: 5'-GTC GAT GGA TGA CTG CTG C, 5'-CAT GGG GAC TCA GGA GAT TG;

d14mit95: 5'-TAT TTT TAA GTC AGT ATA CAC ATG CGC, 5'-TTA TCC AAG TGT ATT TAA AGA AGA GGC;

d4mit178: 5'-GCC CTG AAG GTA AAT CAG TAA CT, 5'-GCT CAG GAG GTA CAT TGC CT;

d4mit76: 5'-TGA AGG AAC CTG AAG CAA GG, 5'-ACC TCC CAG GAG TGT CCA G;

d4mit233: 5'-TGG TCA TGT GTG TCC ATG C, 5'-ACT TCA TGT AGC CAG GTG GG;

d2mit37: 5'-TGT GCA AGC CAG AAA AGT TG, 5'-GAA GGG GAT TGT AAA TTG GTA CC;

d2mit164: 5'-TCT CTG CTA ATT AAG TTG AAG AGT GC, 5'-ACC AGT GTG TGT TTG TAT GAT GTG;

d2mit504: 5'-ATT TCA CAA GTC TTC CCC CC, 5'-TGA AAC ACA AAT GAG CAA CTA CG;

d13mit16: 5'-CCA GCT GAA GGC TTA CTC GT, 5'-AAA GTT AGA ATC AGC CAT TCA AGG;

d13mit61: 5'-TGC TCC AAT ACA ACA AGG TCC, 5'-CCA GCC AAG GTG TGT TGA C;

d13mit9: 5'-GGG TTC CAG ATT GAG TGG AA, 5'-TTG CCA AAG TGT CAA AAT CA;

d5mit391: 5'-AAT AAG AAA ATT CCA CCA AGT CTA CA, 5'-CTT GAT GGG TCT GAT GCC TT;

d5mit69: 5'-CCA GCC TTT CTG GAG TGA AG, 5'-ACC ATG GCA GAA AGC AGT TT.

Supplementary Figures

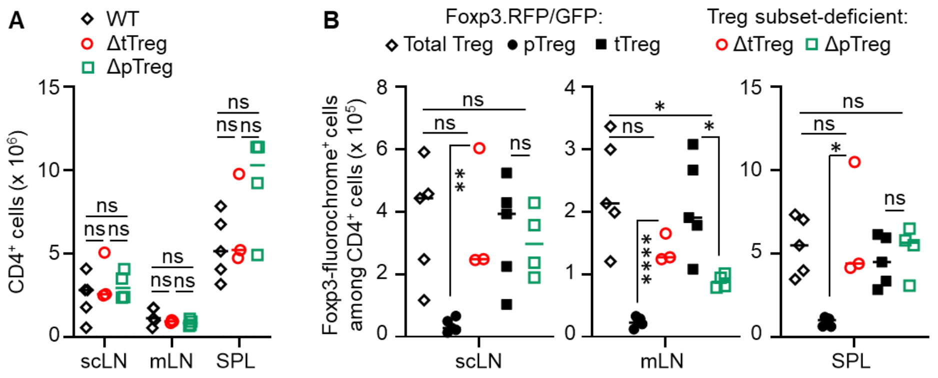

Supplementary Figure S1: Treg cell numbers in peripheral lymphoid tissues. Numbers of **(A)** $CD4^+$ T cells and **(B)** CD4-gated Foxp3-fluorochrome$^+$ Treg cells in subcutaneous lymph nodes (scLNs), mesenteric LNs (mLNs), and spleen (SPL) of $Foxp3^{RFP/GFP}$ mice (pTreg: filled black circles, n = 5; tTreg: filled black squares, n = 5; $CD4^+$ T cells or total Treg: open black diamonds, n = 5), ΔtTreg mice ($CD4^+$ T cells or pTreg: open red circles, n = 3), and ΔpTreg mice ($CD4^+$ T cells or tTreg: open green squares, n = 4). Note that the cell numbers depicted in **(B)** correspond to the percentages shown in **Figure 1E**. Symbols and horizontal lines represent individual mice and mean values, respectively. Unpaired t-test: ns, not significant; $*p \leq 0.05$.

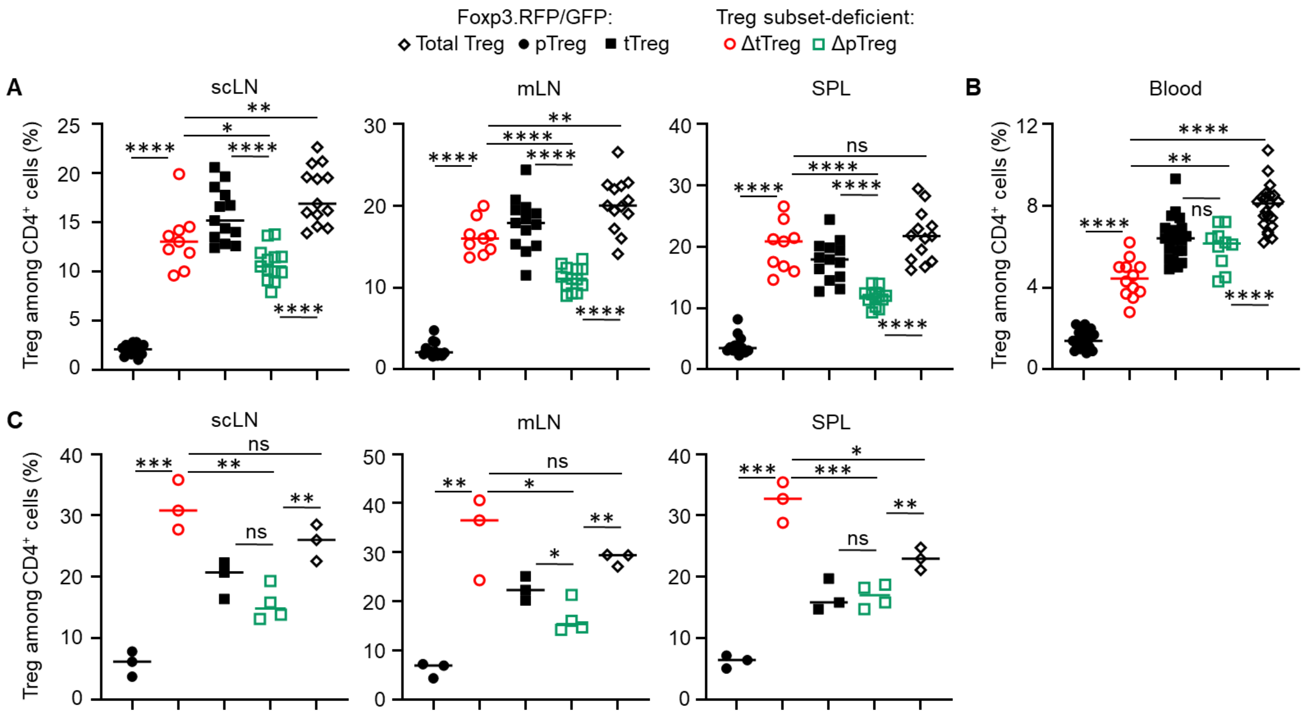

Supplementary Figure S2: Treg cell frequencies in peripheral lymphoid tissues and blood. Percentages of CD4-gated Foxp3-fluorochrome^{+} Treg cells in peripheral lymphoid tissues (scLNs, mLNs, and SPL) and peripheral blood of **(A, B)** 13-22-week-old (13 mice per group) and **(C)** 52-56-week-old (3-4 mice per group) male mice, as indicated. (Foxp3$^{RFP/GFP}$ mice: RFP^{+}GFP$^{–}$ pTreg, filled black circles; RFP^{+}GFP^{+} tTreg: filled black squares; total RFP^{+} Treg: open black diamonds; ΔtTreg mice: RFP^{+}GFP$^{–}$ pTreg, open red circles; ΔpTreg mice: GFP^{+} tTreg, open green squares). Symbols and horizontal lines represent individual mice and mean values, respectively. Unpaired t-test: ns, not significant; *p ≤ 0.05, **p ≤ 0.01, ***p ≤ 0.001, ****p ≤ 0.0001.

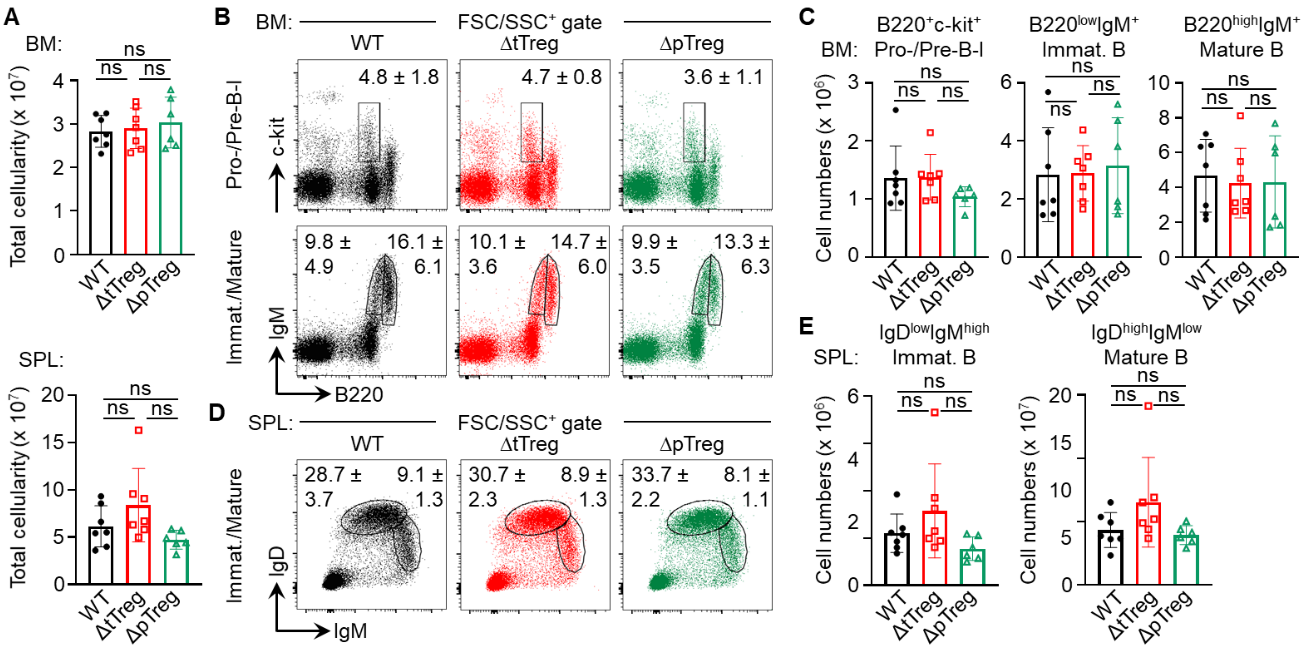

Supplementary Figure S3: Normal B lymphopoiesis in adult ΔtTreg and ΔpTreg mice. Flow cytometry of B cell development in bone marrow (BM) and SPL of ΔtTreg (n = 7), ΔpTreg (n = 6), and Foxp3$^{RFP/GFP}$ (n = 7) mice at 10-15 weeks of age. **(A)** Total cellularity of BM (top) and SPL (bottom). **(B-E)** B cell development. **(B)** Representative flow cytometry and **(C)** numbers of B220^{+}c-kit^{+} Pro/Pre-B-I cells (top), as well as immature B220^{low}IgM^{+} and mature/recirculating B220^{high}IgM^{+} B cells (bottom) in BM. **(D)** Representative flow cytometry and **(E)** numbers of immature IgD^{low}IgM^{high} (left) and mature IgD^{high}IgM^{low} (right) B cells in SPL. Numbers in dot plots in **(B, D)** represent mean percentages of cells ± SD within the respective gate. Symbols and bars in **(A, C, E)** represent individual mice (WT: black circles; ΔtTreg: open red squares; ΔpTreg: open green triangles) and mean values, respectively. Unpaired t-test: ns, not significant. Data are from two independent experiments with 6-7 mice per group.

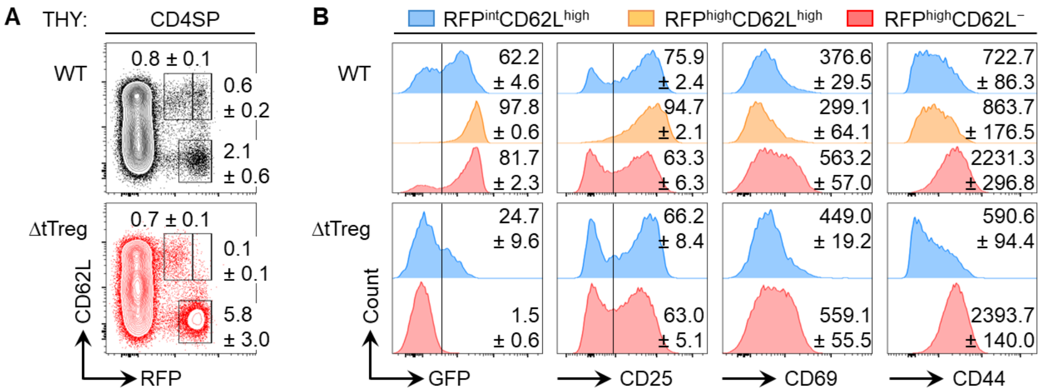

Supplementary Figure S4: pTreg cells recirculate to the thymus of ΔtTreg mice. Tracking thymic recirculation of mature Treg cells to the thymus. **(A)** Representative dot plots of CD62L and Foxp3-driven RFP reporter expression among CD4SP-gated cells in the thymus of Foxp3$^{RFP/GFP}$ (WT, n = 5) and ΔtTreg (n = 3) males at 17-22 weeks of age. **(B)** Histograms show the expression of GFP and surface markers on gated cell populations, as indicated (blue: RFP^{int}CD62L^{high}; yellow: RFP^{high}CD62L^{high}; red: RFP^{high}CD62L$^{–}$). Note that the RFP^{high}CD62L^{high} CD4SP compartment is readily detectable in Foxp3$^{RFP/GFP}$ mice (top panels) but below the detection limit in ΔtTreg mice (bottom panels) due to selective DTA-mediated deletion. Numbers in dot plots in **(A)** and histograms of GFP and CD25 expression in **(B)** represent mean percentages of cells ± SD within the respective gate. Numbers in histograms of CD69 and CD44 expression represent mean fluorescence intensities (MFI) of marker expression ± SD. Data are from a single experiment representative of 3 experiments performed (3-6 mice per experiment).

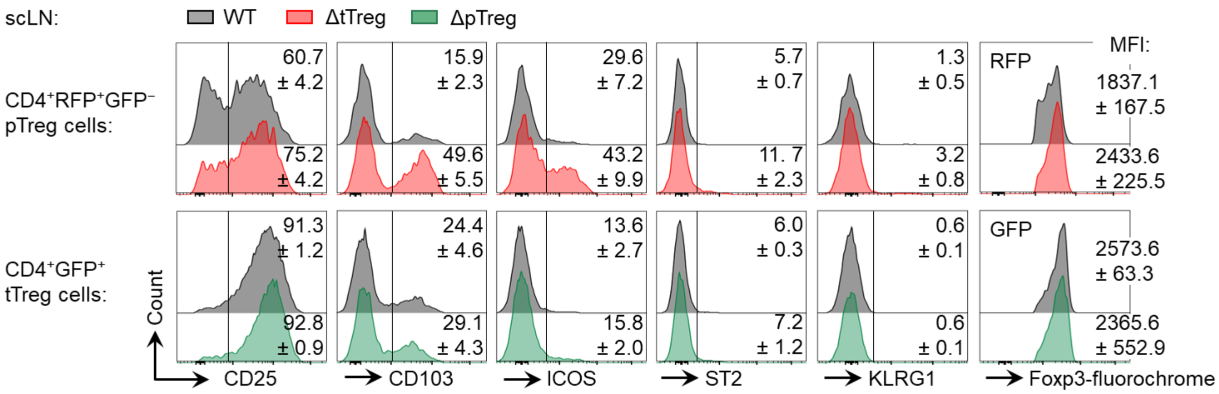

Supplementary Figure S5: Flow cytometric analysis of signature protein expression. Expression analysis of selected Treg cell signature proteins and Foxp3-fluorochrome reporters on gated Treg cell subsets from scLNs of Foxp3$^{RFP/GFP}$ littermates (WT, grey histograms), ΔtTreg mice (red histograms, top panels), and ΔpTreg mice (green histograms, bottom panels), corresponding to data depicted in **Figure 6D**. Numbers in histograms indicate mean percentages of cells ± SD within the respective gate, with the exception of Foxp3 showing mean fluorescence intensity (MFI) of the fluorochrome reporters (RFP, GFP).

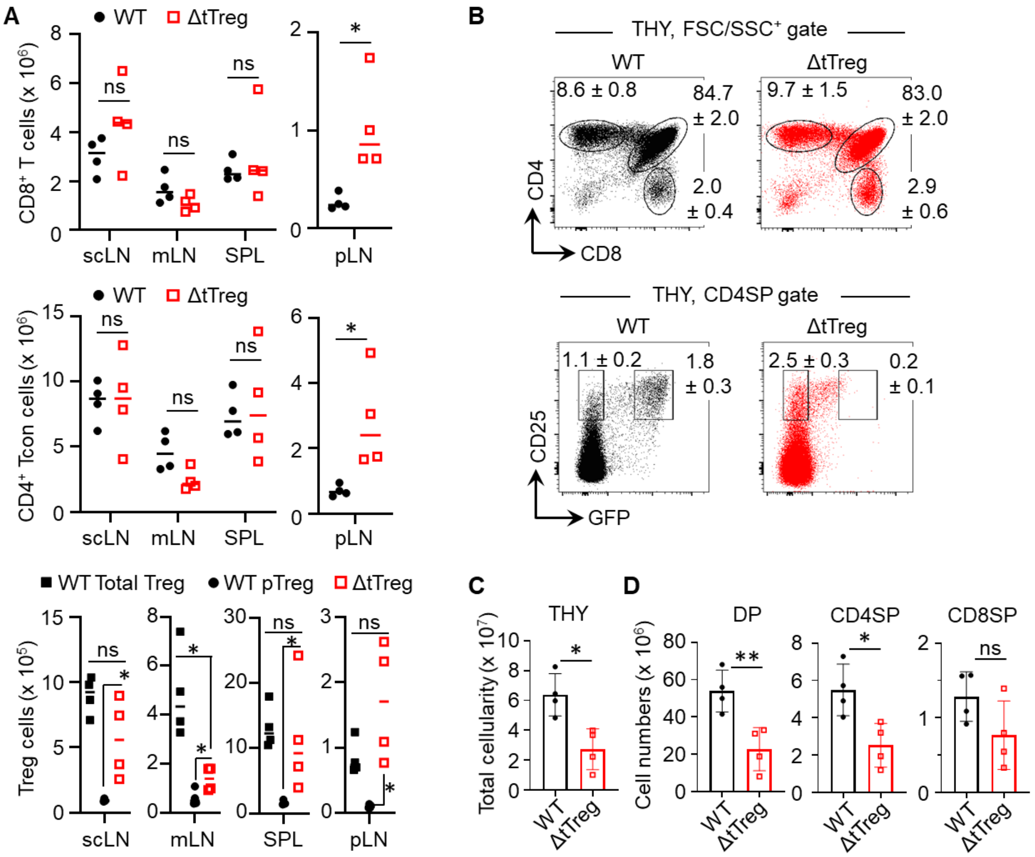

Supplementary Figure S6: Peripheral T cell numbers and intrathymic tTreg cell ablation in (B6>NOD)F2 ΔtTreg mice. (A) Numbers of CD8$^+$ T cells (top), CD4$^+$ T cells (middle) and Treg cells (bottom) from pancreatic LN (pLN, right) and other peripheral lymphoid tissues (scLN, mLN, SPL) of Foxp3$^{RFP/GFP}$ (WT; CD8$^+$, CD4$^+$ T cells, pTreg: closed black circles; total Treg: closed black squares) and ΔtTreg (open red squares) mice (all 8-week-old I-Ag7$^{+/+}$ males), corresponding to data depicted in **Figure 8B-D. (B-D)** Intrathymic tTreg cell ablation and thymopoiesis. **(B)** Representative flow cytometry of CD4 and CD8 expression (left) and CD25 and Foxp3-driven GFP expression (right) among FSC/SSC-gated cells from the thymus (THY) of 8-week-old Foxp3$^{RFP/GFP}$ and ΔtTreg male mice. Numbers in dot plots indicate mean percentages of cells ± SD within the respective gate. **(C)** Total thymic cellularity, as well as **(D)** numbers of DP, CD4SP, and CD8SP thymocytes, as indicated. Symbols and bars represent individual mice and mean values ± SD, respectively. Data in **(B-D)** are from a single experiment (n = 4), corresponding to the data depicted in **Figures 8B-D**. Unpaired t-test: ns, not significant; *p ≤ 0.05, **p ≤ 0.01.

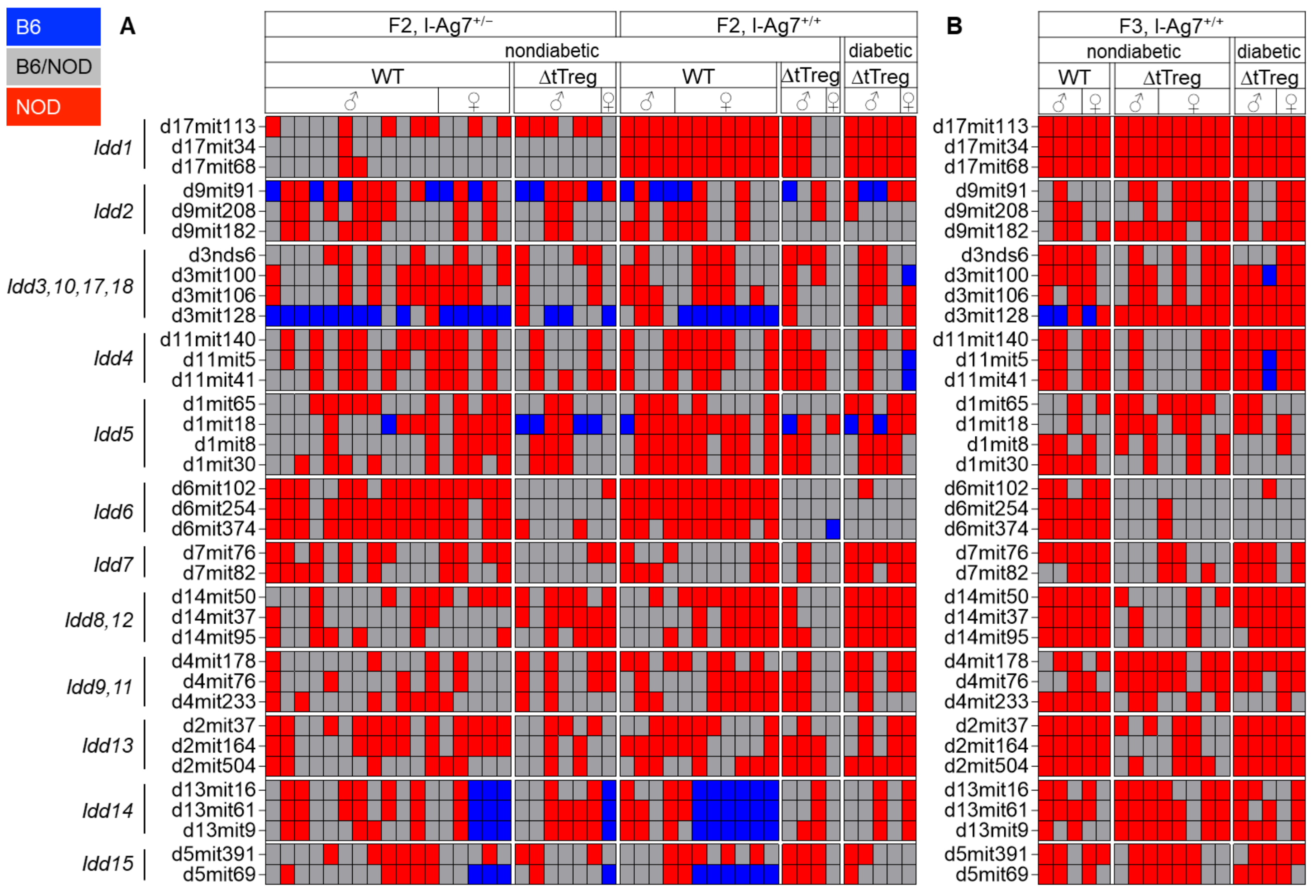

Supplementary Figure S7: Genomic DNA-based *Idd* gene locus analysis of individual (B6>NOD) hybrid mice. Cohorts **(A)** of F2 Foxp3$^{RFP/GFP}$ mice (nondiabetic WT; I-Ag7$^{+/-}$: n = 17; nondiabetic I-Ag7$^{+/+}$, n = 11); F2 ΔtTreg mice (nondiabetic I-Ag7$^{+/-}$, n = 7; nondiabetic, I-Ag7$^{+/+}$, n = 4; diabetic I-Ag7$^{+/+}$, n = 5), and **(B)** of F3 Foxp3$^{RFP/GFP}$ mice (nondiabetic WT; I-Ag7$^{+/+}$, n = 5) and F3 ΔtTreg mice (nondiabetic I-Ag7$^{+/+}$, n = 8; diabetic I-Ag7$^{+/+}$, n = 5) were subjected to genomic PCR for *Idd* gene analysis. The NOD *Idd* status (blue: absent; grey: heterozygous; red: homozygous) of all 36 loci (including subloci) is shown in individual male (♂) and female (♀) mice for each experimental group, as indicated. Note that the R26-DTA transgene of ΔtTreg mice is embedded within the *Idd6* gene locus, resulting in a marked underrepresentation of *Idd6* in F2 and F3 ΔtTreg mice.
